# Syntheses, characterizations, crystal structures and Hirshfeld surface analyses of methyl 4-[4-(di­fluorometh­oxy)phen­yl]-2,7,7-trimethyl-5-oxo-1,4,5,6,7,8-hexa­hydro­quinoline-3-carboxyl­ate, isopropyl 4-[4-(di­fluoro­meth­oxy)phen­yl]-2,6,6-trimethyl-5-oxo-1,4,5,6,7,8-hexa­hydro­quinoline-3-carboxyl­ate and *tert*-butyl 4-[4-(di­fluoro­meth­oxy)phen­yl]-2,6,6-trimethyl-5-oxo-1,4,5,6,7,8-hexa­hydro­quinoline-3-carboxyl­ate

**DOI:** 10.1107/S2056989024001233

**Published:** 2024-02-08

**Authors:** Sema Öztürk Yıldırım, Mehmet Akkurt, Ezgi Pehlivanlar, Gökalp Çetin, Rahime Şimşek, Ray J. Butcher, Ajaya Bhattarai

**Affiliations:** a Department of Physics, Faculty of Science, Eskisehir Technical University, Yunus Emre Campus 26470 Eskisehir, Türkiye; b Department of Physics, Faculty of Science, Erciyes University, 38039 Kayseri, Türkiye; c Department of Physics, Faculty of Sciences, Erciyes University, 38039 Kayseri, Türkiye; d Department of Pharmaceutical Chemistry, Faculty of Pharmacy, Hacettepe University, 06100 Sıhhiye-Ankara, Türkiye; e Department of Pharmaceutical Chemistry, Faculty of Pharmacy, Erzincan Binali Yıldırım University, 24100 Erzincan, Türkiye; f Department of Chemistry, Howard University, Washington DC 20059, USA; g Department of Chemistry, M.M.A.M.C (Tribhuvan University), Biratnagar, Nepal; Texas A & M University, USA

**Keywords:** crystal structure, 1,4-di­hydro­pyridine ring, cyclo­hexene ring, quinoline ring system, disorder, van der Waals inter­actions, Hirshfeld surface analysis

## Abstract

The crystal structures and Hirshfeld surface analyses of three similar compounds are reported. Methyl 4-[4-(di­fluoro­meth­oxy)phen­yl]-2,7,7-trimethyl-5-oxo-1,4,5,6,7,8-hexa­hydro­quinoline-3-carboxyl­ate, (C_21_H_23_F_2_NO_4_), (**I**), crystallizes in the monoclinic space group *C*2/*c* with *Z* = 8, while isopropyl 4-[4-(di­fluoro­meth­oxy)phen­yl]-2,6,6-trimethyl-5-oxo-1,4,5,6,7,8-hexa­hydro­quinoline-3-carb­oxyl­ate, (C_23_H_27_F_2_NO_4_), (**II**) and *tert*-butyl 4-[4-(di­fluoro­meth­oxy)phen­yl]-2,6,6-trimethyl-5-oxo-1,4,5,6,7,8-hexa­hydro­quinoline-3-carboxyl­ate, (C_24_H_29_F_2_NO_4_), (**III**) crystallize in the ortho­rhom­bic space group *Pbca* with *Z* = 8. In the crystal structure of (**I**), mol­ecules are linked by N—H⋯O and C—H⋯O inter­actions, forming a tri-periodic network, while mol­ecules of (**II**) and (**III**) are linked by N—H⋯O, C—H⋯F and C—H⋯π inter­actions, forming layers parallel to (002). The cohesion of the mol­ecular packing is ensured by van der Waals forces between these layers. In (**I**), the atoms of the 4-di­fluoro­meth­oxy­phenyl group are disordered over two sets of sites in a 0.647 (3): 0.353 (3) ratio. In (**III**), the atoms of the dimethyl group attached to the cyclo­hexane ring, and the two carbon atoms of the cyclo­hexane ring are disordered over two sets of sites in a 0.646 (3):0.354 (3) ratio.

## Abstract 2

In the crystal structure of methyl 4-[4-(di­fluoro­meth­oxy)phen­yl]-2,7,7-trimethyl-5-oxo-1,4,5,6,7,8-hexa­hydro­quinoline-3-carboxyl­ate (**I**), mol­ecules are linked by N—H⋯O and C—H⋯O inter­actions, forming a tri-periodic network, while mol­ecules of isopropyl 4-[4-(di­fluoro­meth­oxy)phen­yl]-2,6,6-trimethyl-5-oxo-1,4,5,6,7,8-hexa­hydro­quinoline-3-carboxyl­ate (**II**) and *tert*-butyl 4-[4-(di­fluoro­meth­oxy)phen­yl]-2,6,6-trimethyl-5-oxo-1,4,5,6,7,8-hexa­hydro­quinoline-3-carboxyl­ate (**III**) are linked by N—H⋯O, C—H⋯F and C—H⋯π inter­actions, forming layers parallel to (002).

## 1. Chemical context

Inflammation is a defense tool developed by the immune system to eliminate abnormal conditions resulting from harmful stimuli caused by pathogens, damaged cells, toxic compounds and traumatic cells. Inflammatory processes are important in terms of providing hemostasis of the body. Inflammatory mediators such as cytokines, chemokines and leukocytes secreted by the immune system during inflammation regulate the vital functions of the cell such as survival, growth and proliferation. In some cases, persistent and uncontrolled acute inflammatory responses cause chronic inflammation (Chen *et al.*, 2018 ▸; Aqdas & Sung, 2023 ▸).

Cancer is a dangerous disease with a high incidence all over the world. Although chemotherapy, radiotherapy and surgical inter­ventions are among the current treatment methods, there are cases where these methods are insufficient. In addition, cancer is a disease that progresses rapidly and can recur even after treatment. Therefore, there is an urgent need for new treatments and new therapeutic agents (Shaheen *et al.*, 2020 ▸). Tumor tissues are formed by the abnormal and damaged proliferation of cancer cells. Inflammation mediators multiply uncontrollably by immune cells in the microenvironment of tumor tissue (Aqdas & Sung, 2023 ▸). This uncontrolled development of inflammation is the root cause of many chronic diseases and cancers. Therefore, it is very important to develop new anti-inflammatory treatments (Wu *et al.*, 2022 ▸).

**Figure 1:**
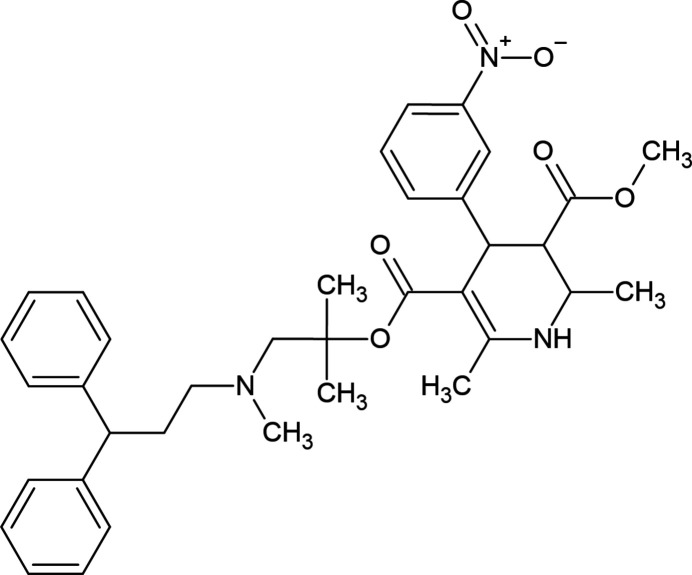
Structure of lercanidipine

1,4-DHPs and their condensed derivatives are heterocyclic compounds with many pharmacological and biological activities. These compounds were described in the literature for the first time with their calcium channel modulator activities, and then various activities such as anti­cancer and anti-ischemic were discovered (Bryzgalov *et al.*, 2023 ▸). Lerkadipine, which is a calcium channel blocker in the pharmaceutical market, has also been shown by *in vivo* studies to be effective in melanoma and non-small-cell lung cancer. Based on this information, new compounds with anti-inflammatory effects have been obtained with modifications made on 1,4-DHPs and their activities have been proven (Pan *et al.*, 2022 ▸) (Fig. 1 ▸). Hexa­hydro­quinolines are heterocyclic rings obtained by the condensation of 1,4-DHPs with the cyclo­hexane ring. In recent years, it has been seen that hexa­hydro­quinoline derivatives have many biological activities such as analgesic, anti­cancer, anti­bacterial, anti­tuberculosis, anti­malarial, anti­oxidant, anti-inflammatory, anti-Alzheimer’s. Therefore, the hexa­hydro­quinoline ring system is a very well-established motif for medicinal chemistry and has been the subject of many studies in recent years (Ranjbar *et al.*, 2019 ▸). 
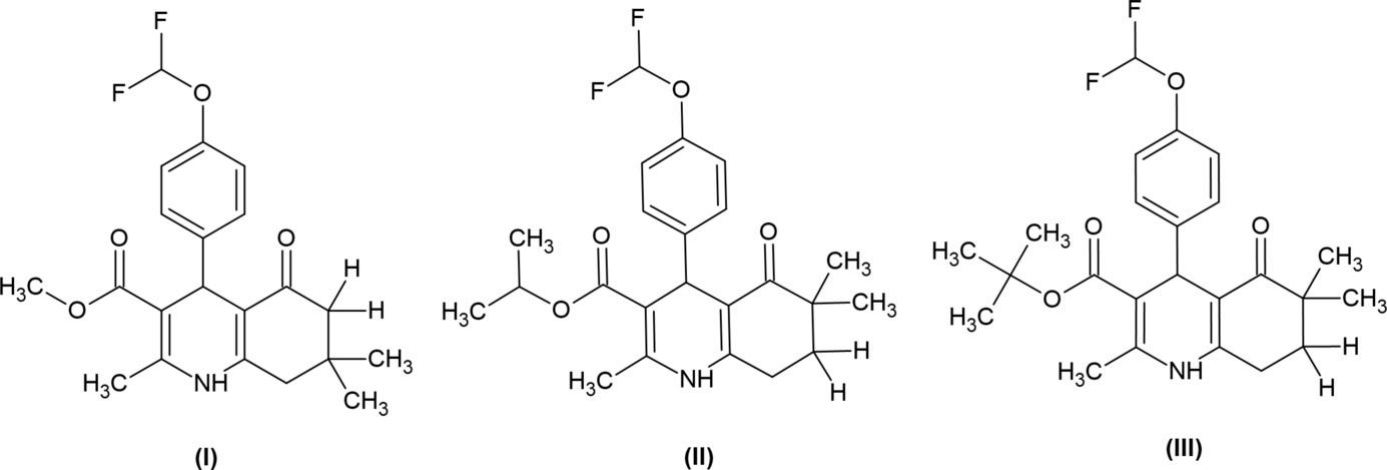


## 2. Structural commentary

**Figure 2:**
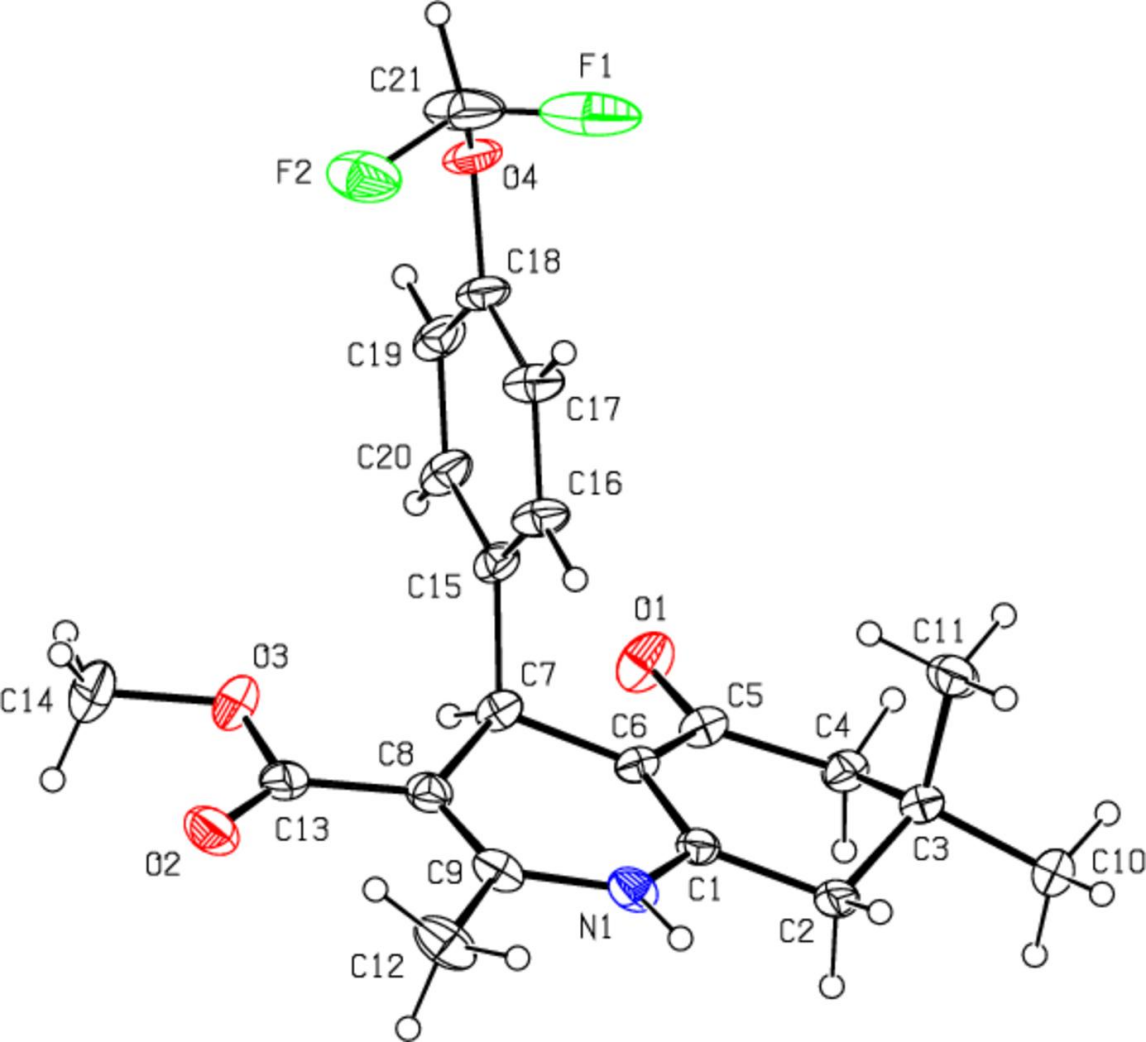
The mol­ecular structure of (**I**) with displacement ellipsoids drawn at the 30% probability level. Only the major component of disorder is shown for clarity.

The 1,4-di­hydro­pyridine ring (N1/C1/C6–C9) of compound (**I**) (Fig. 2 ▸) adopts a distorted boat conformation [puckering parameters (Cremer & Pople, 1975 ▸) are *Q*_T_ = 0.196 (3) Å, θ = 72.2 (9)° and φ = 185.8 (8)°], while the cyclo­hexene ring (C1–C6) has a distorted half-chair conformation [puckering parameters are *Q*_T_ = 0.466 (3) Å, θ = 123.1 (4)° and φ = 295.3 (4)°]. The atoms of the 4-di­fluoro­meth­oxy­phenyl group in (**I**) are disordered over two sets of sites with refined occupancy factors of 0.647 (3) and 0.353 (3). The major (C15–C20) and minor (C15*A*–C20*A*) disorder components of the 4-[4-(di­fluoro­meth­oxy]phenyl ring make dihedral angles of 80.84 (15) and 85.81 (27)°, respectively, with the mean plane of the quinoline ring system [N1/C1–C9; maximum deviation = 0.382 (2) Å for C3].

**Figure 3:**
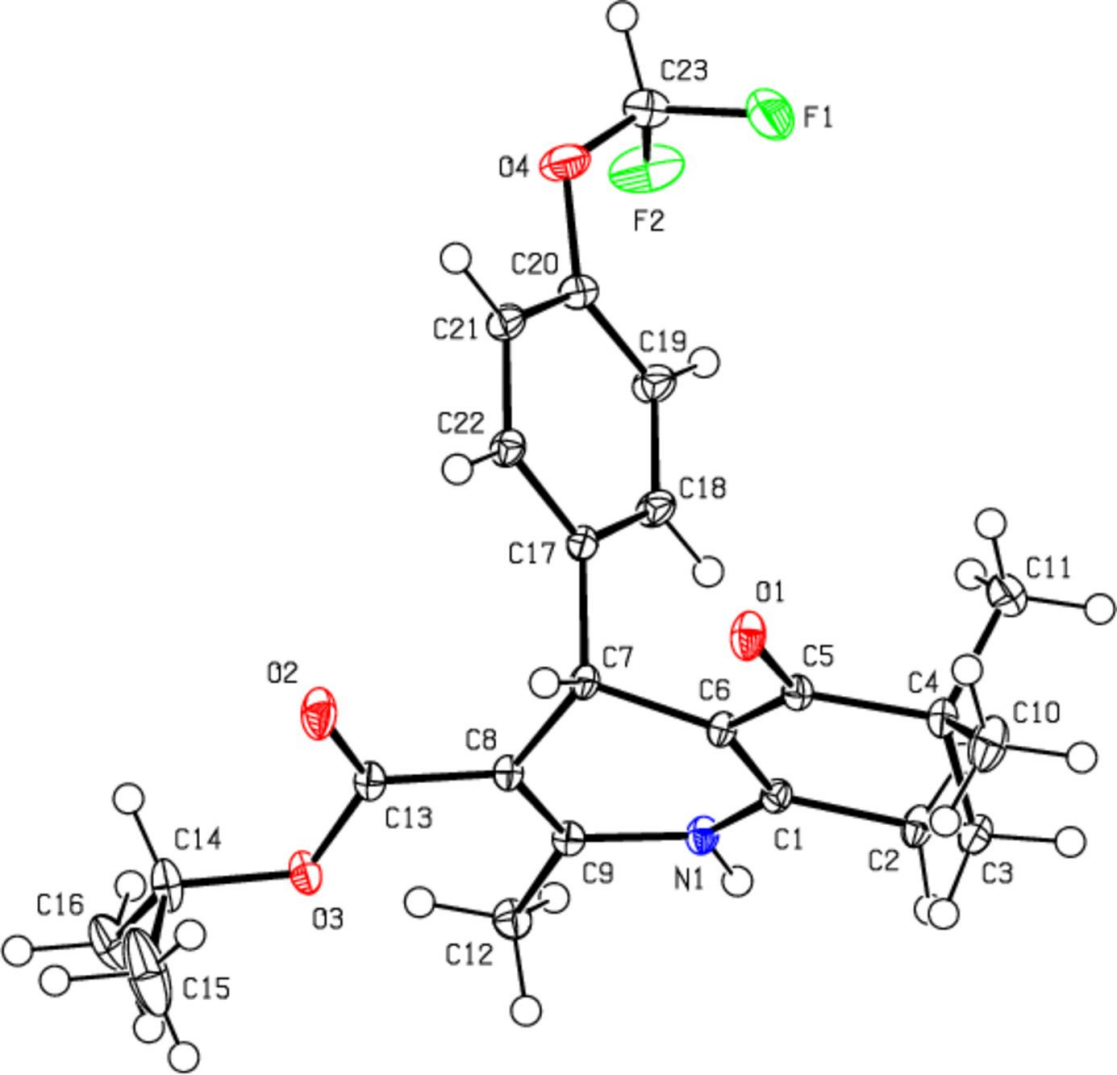
The mol­ecular structure of (**II**) with displacement ellipsoids drawn at the 50% probability level.

In (**II**) (Fig. 3 ▸), the 1,4-di­hydro­pyridine ring (N1/C1/C6–C9) and the cyclo­hexene ring (C1–C6) both have distorted boat conformations [puckering parameters are *Q*_T_ = 0.3187 (9) Å, θ = 105.86 (16)° and φ = 359.72 (17)° for the 1,4-di­hydro­pyridine ring, and *Q*_T_ = 0.4332 (11) Å, θ = 131.14 (13)° and φ = 301.37 (17)° for the cyclo­hexene ring]. The 4-[4-(di­fluoro­meth­oxy]phenyl ring (C17–C22) makes a dihedral angle of 86.39 (4)° with the mean plane of the quinoline ring system [N1/C1–C9; maximum deviation = 0.421 (1) Å for C3].

**Figure 4:**
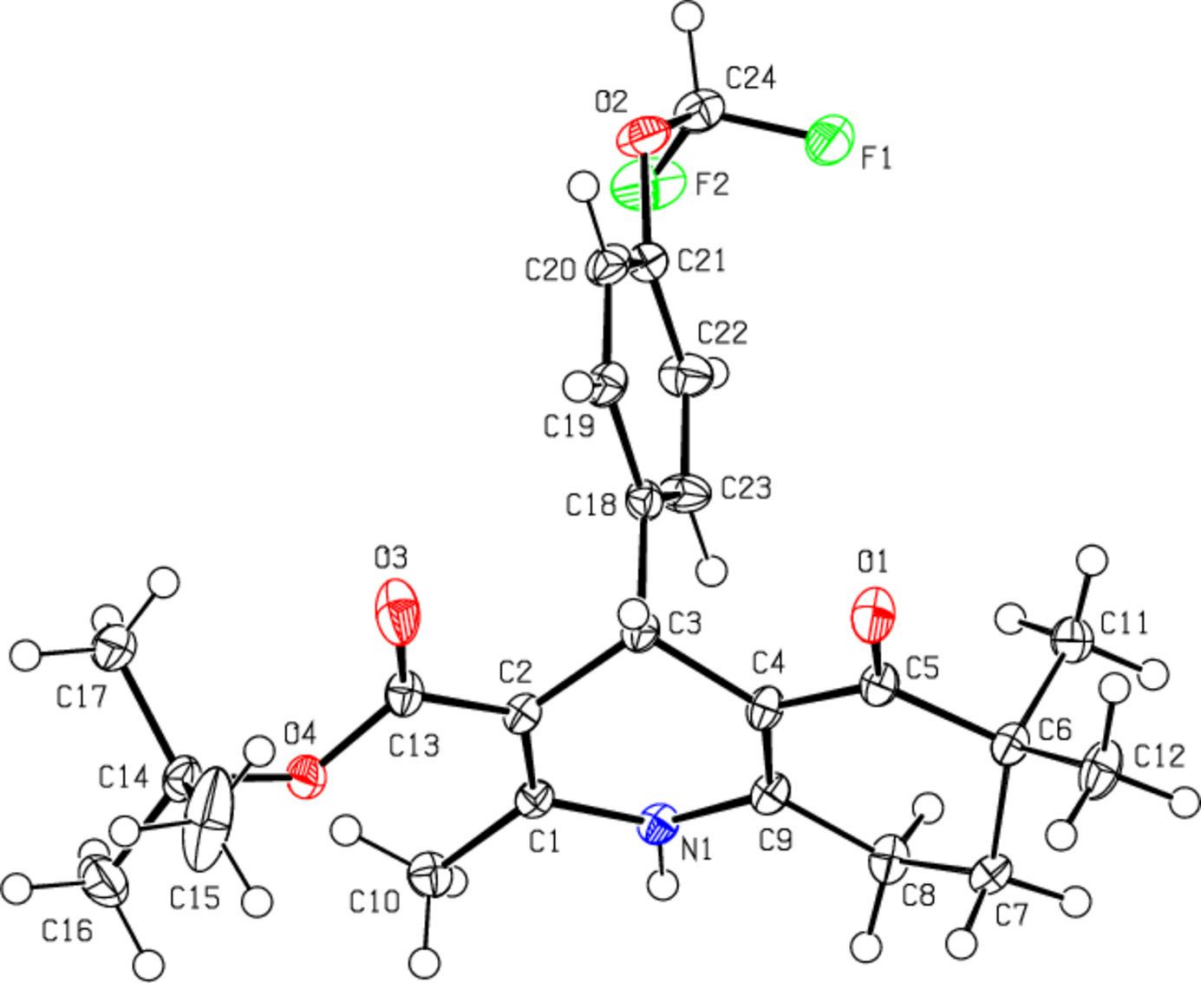
The mol­ecular structure of (**III**) with displacement ellipsoids drawn at the 50% probability level. Only the major component of disorder is shown for clarity.

In (**III**) (Fig. 4 ▸), the 1,4-di­hydro­pyridine ring (N1/C1–C4/C9) and the cyclo­hexene ring (C4–C9) both have distorted boat conformations [puckering parameters are *Q*_T_ = 0.3403 (14) Å, θ = 73.4 (2)° and φ = 180.4 (3)° for the 1,4-di­hydro­pyridine ring, and *Q*_T_ = 0.420 (5) Å, θ = 131.7 (6)° and φ = 356.2 (10)° for the cyclo­hexene ring]. The two carbon atoms (C7/C7*A* and C8/C8*A*) in the cyclo­hexane ring of the quinoline ring system are disordered over two sets of sites in a 0.646 (3):0.354 (3) ratio. The 4-[4-(di­fluoro­meth­oxy]phenyl ring (C18–C23) makes dihedral angles of 84.47 (4) and 88.71 (5)°, respectively, with the mean planes of the major and minor disorder components of the quinoline ring system [N1/C1–C9; maximum deviation = −0.427 (3) Å for C7 in the major component and N1/C1–C6/C7*A*/C8*A*/C9; maximum deviation = 0.392 (3) Å for C3 in the minor component].

Bond lengths and angles in all compounds are in agreement with those reported for the related compounds discussed in the *Database survey* section.

## 3. Supra­molecular features and Hirshfeld surface analysis

**Figure 5:**
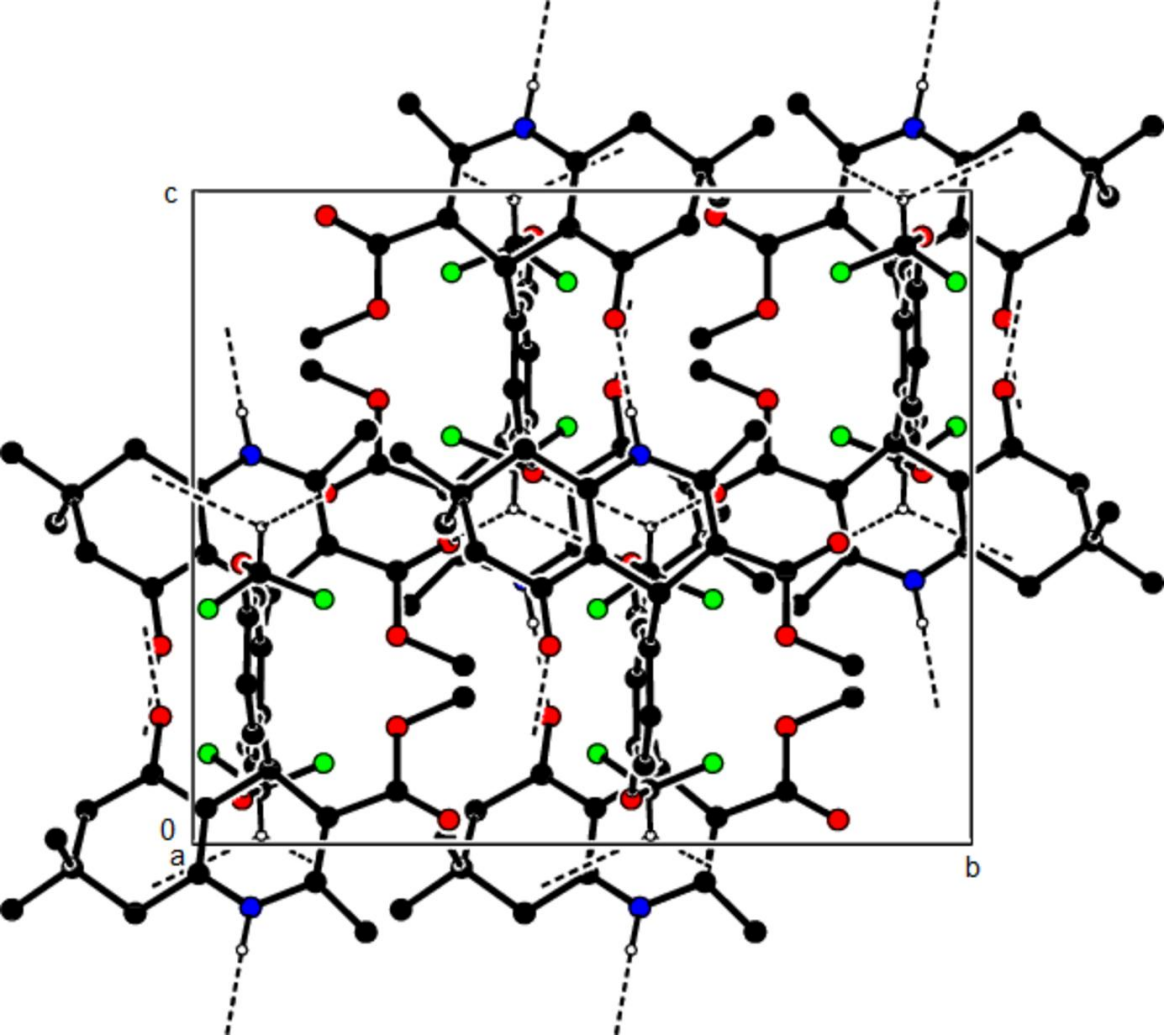
The N—H⋯O and C—H⋯O contacts (solid lines) of (**I**), shown along the *a*-axis. Only the major component of disorder is shown for clarity.

**Figure 8:**
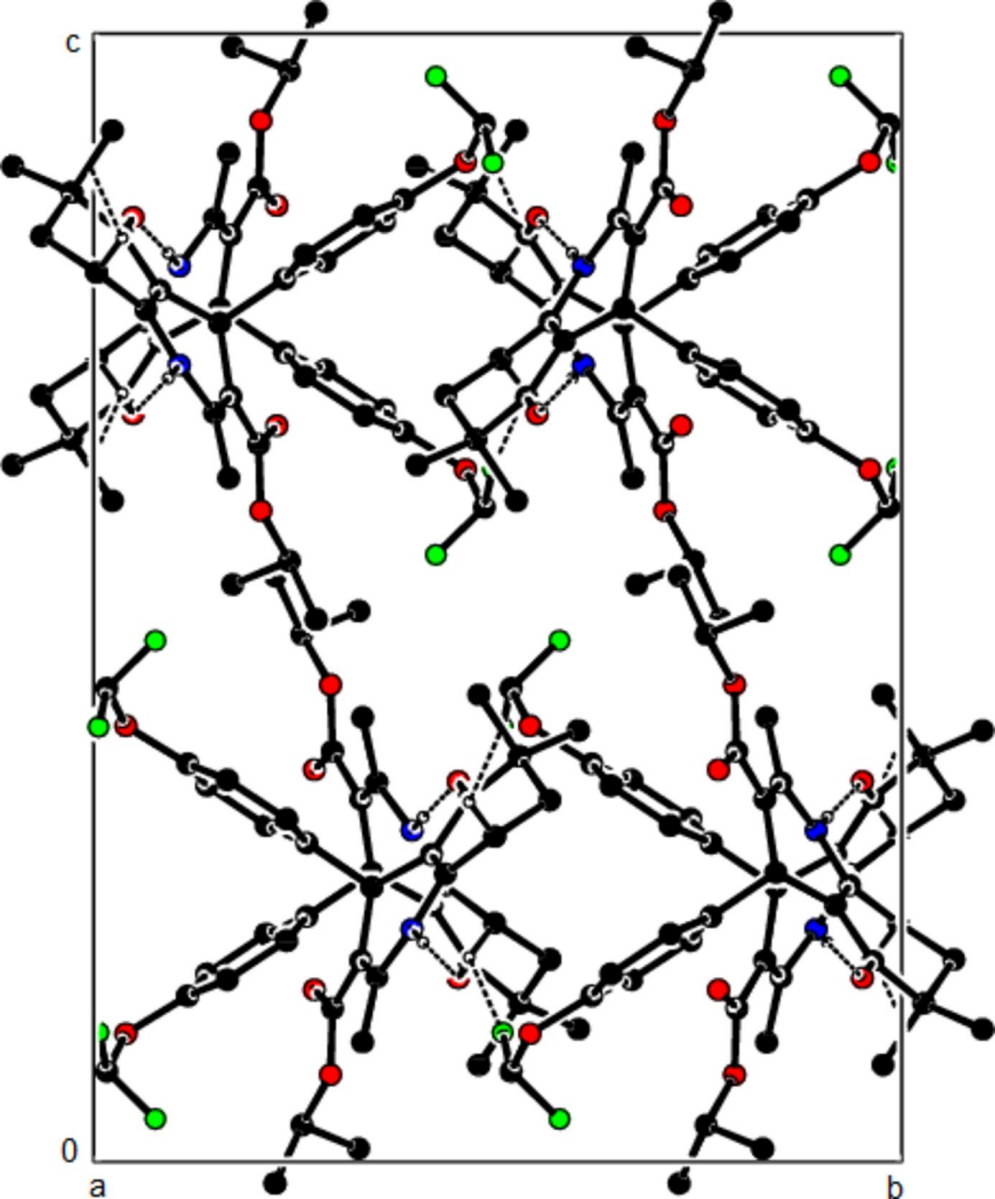
The N—H⋯O and C—H⋯F contacts (solid lines) of (**II**), shown along the *a*-axis.

**Figure 12:**
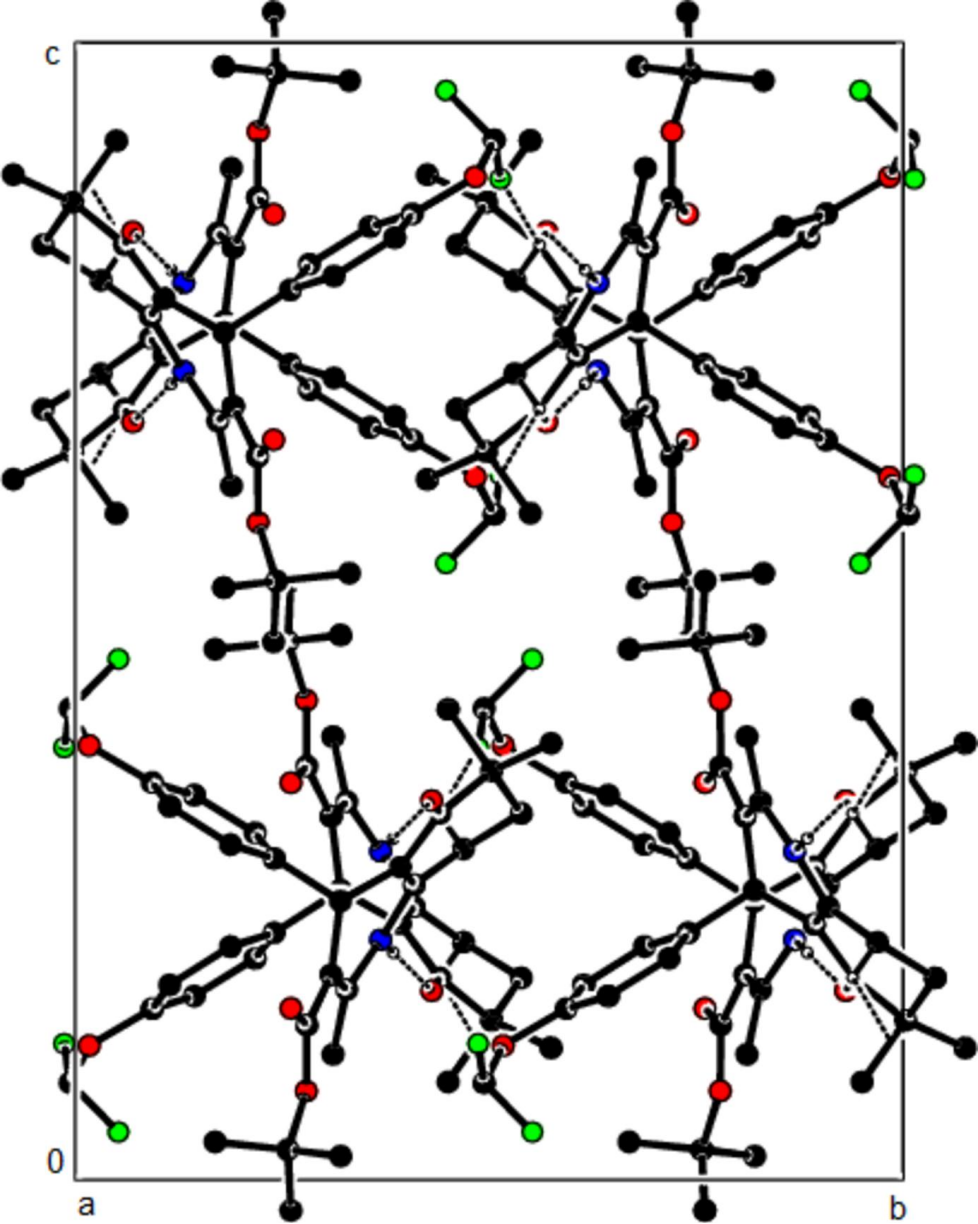
The N—H⋯O and C—H⋯F contacts (solid lines) of (**III**), shown along the *a*-axis. Only the major component of disorder is shown for clarity.

In the crystal structure of (**I**), mol­ecules are linked by N—H⋯O and C—H⋯O inter­actions, forming a tri-periodic network (Table 1 ▸; Figs. 5 ▸, 6 ▸ and 7 ▸), while mol­ecules of (**II**) and (**III**) are linked by N—H⋯O, C—H⋯F and C—H⋯π inter­actions, forming layers parallel to (002) [Table 2 ▸, Figs. 8 ▸, 9 ▸, 10 ▸ and 11 ▸; C3—H3*B*⋯*Cg*3^*a*^: H3*B*⋯*Cg*3^*a*^ = 3.6716 (14) Å, C3—H3*B*⋯*Cg*3^*a*^ = 158°; symmetry code: (*a*) 1 − *x*, 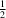 + *y*, 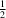 − *z*; *Cg*3 is the centroid of the 4-di­fluoro­meth­oxy­phenyl ring (C17–C22) for (**II**), and Table 3 ▸, Figs. 12 ▸, 13 ▸, 14 ▸ and 15 ▸; C7—H7*B*⋯*Cg*4^*b*^: H7*B*⋯*Cg*4^*b*^ = 3.687 (2) Å, C7—H7*B*⋯*Cg*4^*b*^ = 158°; symmetry code: (*b*) 1 − *x*, − 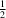 + *y*, 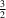 − *z*; *Cg*4 is the centroid of the 4-di­fluoro­meth­oxy-phenyl ring (C18–C23) for (**III**)]. The cohesion of the mol­ecular packing is ensured by van der Waals forces between these layers.

**Figure 16:**
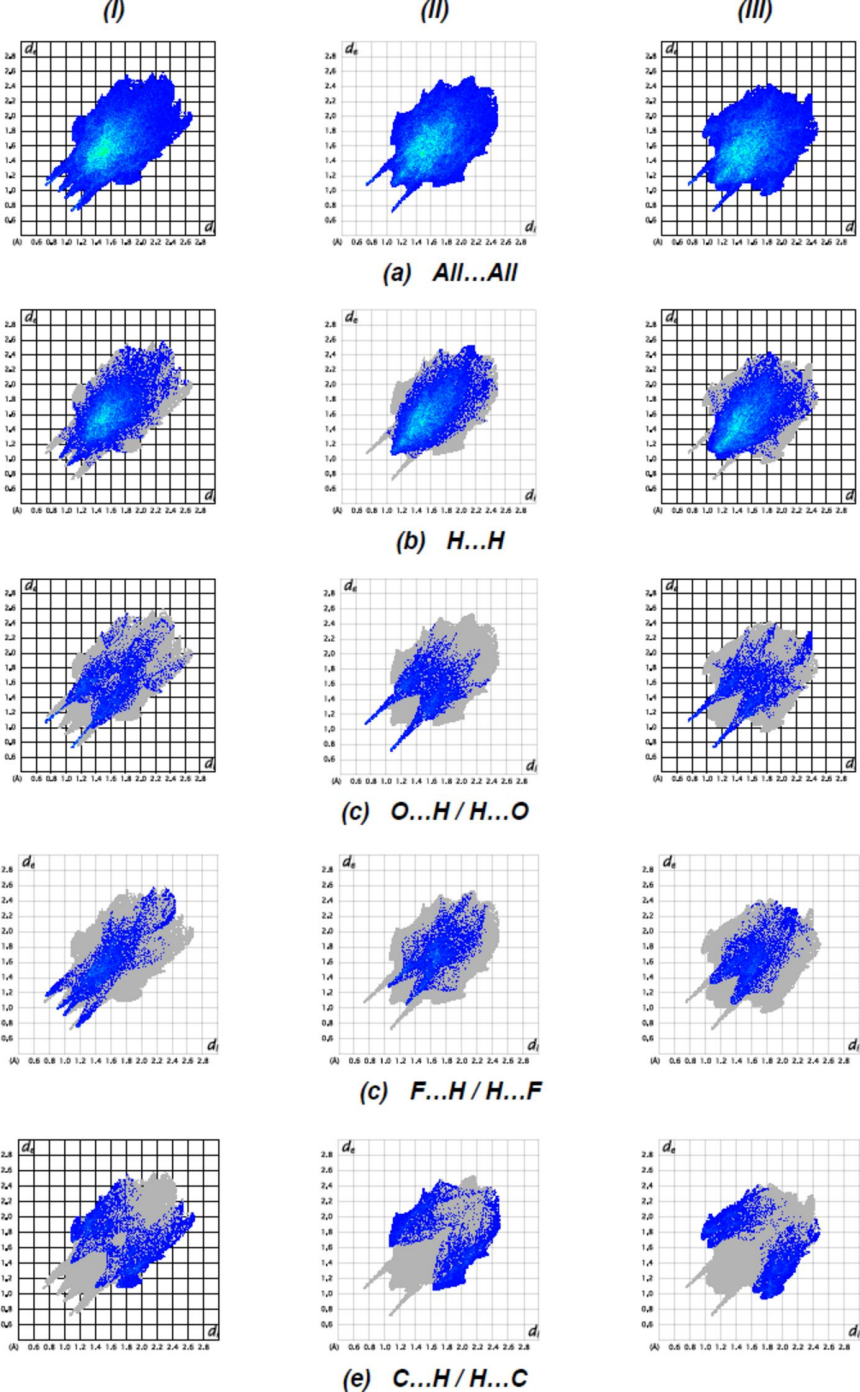
Two-dimensional fingerprint graphs showing the H⋯H, O⋯H/H⋯O, F⋯H/H⋯F and C⋯H/H⋯C inter­actions of (**I**), (**II**) and (**III**).

To qu­antify the inter­molecular inter­actions between the mol­ecules of (**I**), (**II**) and (**III**) in their respective crystal structures, the Hirshfeld surfaces and their corresponding two-dimensional fingerprint plots were calculated using the software package *Crystal Explorer 17.5* (Spackman *et al.*, 2021 ▸). The two-dimensional fingerprint plots are shown in Fig. 16 ▸. The dominant inter­actions of all compounds are H⋯H [(**I**): 49.1%, (**II**): 55.5% and (**III**): 58.9%], O⋯H/H⋯O [(**I**): 17.5%, (**II**): 14.9% and (**III**): 12.7%], F⋯H/H⋯F [(**I**): 16.2%, (**II**): 14.1% and (**III**): 12.9%] and C⋯H/H⋯C [(**I**) 11.7%, (**II**): 14.5% and (**III**): 12.0% ]. The percentage contributions of inter­atomic contacts calculated for each compound are given in Table 4 ▸. These inter­actions play a crucial role in the overall consolidation of the crystal packing. The presence of different functional groups in the compounds leads to some differences in the remaining weak inter­actions.

## 4. Database survey

A search of the Cambridge Structural Database (CSD, Version 5.42, update of September 2021; Groom *et al.*, 2016 ▸) for similar structures with the 1,4,5,6,7,8-hexa­hydro­quinoline group showed that the nine results most closely related to the title compound are LIMYUF (Pehlivanlar *et al.*, 2023 ▸), WEZJUK (Yıldırım *et al.*, 2023 ▸), ECUCUE (Yıldırım *et al.*, 2022 ▸), LOQCAX (Steiger *et al.*, 2014 ▸), NEQMON (Öztürk Yildirim, *et al.*, 2013 ▸), PECPUK (Gündüz *et al.*, 2012 ▸), IMEJOA (Linden *et al.*, 2011 ▸), PUGCIE (Mookiah *et al.*, 2009 ▸), UCOLOO (Linden *et al.*, 2006 ▸) and DAYJET (Linden *et al.*, 2005 ▸). In all these compounds, mol­ecules are linked by N—H⋯O hydrogen bonds. Furthermore, C—H⋯F hydrogen bonds in LIMYUF, C—H⋯O hydrogen bonds in WEZJUK, ECUCUE, NEQMON, IMEJOA and PUGCIE and C—H⋯π inter­actions in LIMYUF, WEZJUK and ECUCUE were also observed.

## 5. Synthesis and crystallization

**Figure 17:**
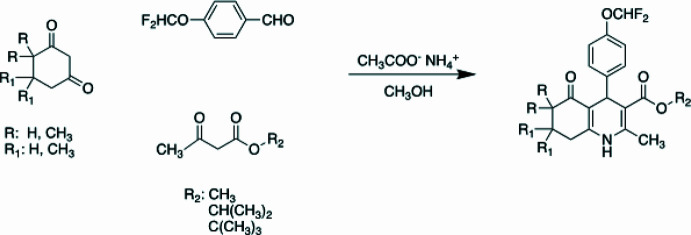
Synthetic scheme

The target compounds were synthesized by 5,5-di­methyl­cyclo­hexane-1,3-dione/4,4-di­methyl­cyclo­hexane-1,3-dione (1 mmol), 4-di­fluoro­meth­oxy­benzaldehyde (1 mmol), methyl aceto­acetate/isopropyl aceto­acetate/*tert*-butyl aceto­acetate (1 mmol), and ammonium acetate (5 mmol), which were refluxed for 8 h in absolute methanol (10 ml). The progress of the reactions were monitored by TLC and after the reactions were seen to be complete, they were cooled to room temperature. The obtained precipitates were filtered and recrystallized from methanol for further purification. The synthetic route is shown in Fig. 17 ▸. The structures of the compounds were elucidated by IR, ^1^H-NMR, ^13^C-NMR and HRMS analysis.

*Methyl 4-[4-(di­fluoro­meth­oxy)phen­yl]-2,7,7-trimethyl-5-oxo-1,4,5,6,7,8-hexa­hydro­quinoline-3-carboxyl­ate (**I**):* Yield: 59%; Yellow solid; mp: 478–479 K; IR (ν, cm^−1^) 3208 (N—H stretching); 3076 (C—H stretching, aromatic); 2956 (C—H stretching, aliphatic); 1700 (C=O stretching, ester); 1649 (C=O stretching, ketone). ^1^H NMR (500 MHz, DMSO-*d*_6_, ppm): δ 0.84 (3H; *s*; 7-CH_3_), 1.00 (3H; *s*; 7-CH_3_), 1.98 (1H; *d*; *J* = 16,05; kinolin H8a), 2.17 (1H; *d*; *J* = 16.05 Hz; quinoline H8b), 2.29 (3H; *s*; 2-CH_3_), 2.29 (1H; *d*; *J* = 16.05 Hz quinoline H6a), 2.30 (2H; *d*; *J* = 16.05 Hz; quinoline H6b), 3.53 (3H; *s*; COOCH_3_), 4.86 (H; *s*; quinoline H4), 6.99 (2H; *d*; *J* = 8.6 Hz; Ar-H3, Ar-H5), 7.13 (1H; *t*; *J* = 74.4 Hz; OCHF_2_), 7.17 (2H; *d*; *J* = 8.6 Hz; Ar-H2, Ar-H6), 9.14 (1H; *s*; NH). ^13^C NMR (125 MHz, DMSO-*d*_6_, ppm): δ 18.8 (2-CH_3_), 26.9 (7-CH_3_), 29.5 (C-7), 32.6 (C-8), 35.6 (C-4), 50.6 (C-6), 51.1 (COOCH_3_), 103.4 (C-3), 110.2 (C-4a), 114.8 (C_3_′), 116.9, 118.6, 118.9 (OCHF_2_), 129.2 (C_2_′), 145.0 (C_1_′), 145.9 (C-2), 149.4 (C-8a), 150.06 (C_4_′), 167.6 (COOCH_3_), 194.7 (C-5). HRMS (ESI/Q-TOF): *m*/*z* calculated for C_21_H_23_F_2_NO_4_ [*M* + H]^+^, 392,1673; found 392.1825.

*Isopropyl 4-[4-(di­fluoro­meth­oxy)phen­yl]-2,6,6-trimethyl-5-oxo-1,4,5,6,7,8-hexa­hydro­quinoline-3-carboxyl­ate (**II**):* Yield: 37%; White solid; mp: 486–487 K; IR (ν, cm^−1^) 3194 (N—H stretching); 2970 (C—H stretching, aromatic); 2939 (C—H stretching, aliphatic); 1674 (C=O stretching, ester). ^1^H NMR (400 MHz, DMSO-*d*_6_, ppm): δ 0.86 (3H; *s*; 6-CH_3_), 0.96 (3H; *s*; 6-CH_3_), 1.0 [3H; *d*; *J* = 6.4 Hz; COOCH(CH_3_)_2a_], 1.15 [3H; *d*; *J*=6.4 Hz; COOCH(CH_3_)_2b_], 1.67–1.70 (2H; *m*; quinoline H7), 2.44 (3H; *m*; quinoline H8), 2.24 (3H; *s*; 2-CH_3_), 4.77–4.82 [1H; *m*; COOCH(CH_3_)_2_], 4.81 (1H; *s*; quinoline H4), 6.95 (2H; *d*; *J* = 8 Hz; Ar-H3) 7.09 (1H; *t*; *J* = 74.4 Hz; OCHF_2_), 7.14 (2H; *d*; *J* = 8 Hz; Ar-H2), Ar-H6, 9.01 (1H; *s*; NH). ^13^C NMR (100 MHz, DMSO-*d*_6_, ppm): δ 18.2 (2-CH_3_), 21.5 [COOCH(CH_3_)_2a_], 21.8 [COOCH(CH_3_)_2b_], 22.8 (C-8), 24.0 (6-CH_3_), 25.0 (C-7), 34.0 (C-4), 35.5 (C-6), 66.0 [COOCH(CH_3_)_2_] 103.3 (C-3), 108.9 (C-4a), 113.8 (C_3_′), 116.6, 118.0, 118.9 (OCHF_2_), 128.8 (C_2_′), 144.7 (C_1_′), 144.9 (C-2), 149.3 (C-8a), 149.7 (C_4_′), 166.2 [COOCH(CH_3_)_2_], 199.3 (C-5). HRMS (ESI/Q-TOF): *m*/*z* calculated for C_23_H_27_F_2_NO_4_ [*M* + H]^+^, 420.1986; found 420.2150.

*tert-Butyl 4-[4-(di­fluoro­meth­oxy)phen­yl]-2,6,6-trimethyl-5-oxo-1,4,5,6,7,8-hexa­hydro­quinoline-3-carboxyl­ate* (**III**): Yield: 20%; White solid; mp: 456–457 K; IR (ν, cm^−1^) 3194 (N—H stretching); 2962 (C—H stretching, aromatic); 2931 (C—H stretching, aliphatic); 1674 (C=O stretching, ester). ^1^H NMR (400 MHz, DMSO-*d*_6_, ppm): δ 0.86 (3H; *s*; 6-CH_3_), 0.95 (3H; *s*; 6-CH_3_), 1.30 [9H; *s*; COOC(CH_3_)_3_], 1.65–1.69 (2H; *m*; quinoline H7), 2.20 (3H; *s*; 2-CH_3_), 2.44–2.47 (2H; *m*; quinoline H8), 4.76 (1H; *s*; quinoline H4), 6.96 (2H; *d*; *J* = 8.4 Hz; Ar-H3, Ar-H5), 7.10 (1H; *t*; *J* = 74.4 Hz; OCHF_2_), 7.13 (2H; *d*; *J* = 8 Hz; Ar-H2, Ar-H6), 8.95 (1H; *s*; NH). ^13^C NMR (100 MHz, DMSO-*d*_6_, ppm): δ 18.1 (2-CH_3_), 22.8 (C-8), 24.0 (6-CH_3_), 25.0 (C-7), 27.8 [COOC(CH_3_)_3_], 34.0 (C-4), 35.7 (C-6), 78.7 [COOC(CH_3_)_3_], 104.4 (C-3), 108.7 (C-4a), 113.8 (C_3_′), 116.3, 118.0, 118.9 (OCHF_2_), 128.7 (C_2_′), 143.9 (C_1_’), 144.9 (C-2), 148.7 (C-8a), 149.7 (C_4_′), 166.3 (COOC(CH_3_)_3_), 199.2 (C-5). HRMS (ESI/Q-TOF): *m*/*z* calculated for C_24_H_29_F_2_NO_4_ [*M* + H]^+^, 434.2143; found 434.2321.

## 6. Refinement

Crystal data, data collection and structure refinement details are summarized in Table 5 ▸. In (**I**), (**II**) and (**III**), the N-bound H atom was located in a difference Fourier map and refined freely [N1—H1*N* = 0.90 (3) Å for (**I**), N1—H1*N* = 0.863 (16) Å for (**II**) and N1—H1*N* = 0.88 (2) Å for (**III**)]. The C-bound H atoms of all compounds were positioned geometrically [C—H = 0.95–1.00 Å] and refined using a riding model with*U*_iso_(H) = 1.2 or 1.5*U*_eq_(C). In (**I**), the atoms of the 4-di­fluoro­meth­oxy-phenyl group are disordered over two sets of sites with refined occupancy factors of 0.647 (3):0.353 (3). In (**III**), the carbon atoms (C10, C13–C24) of the methyl and *tert*-butyl formate group attached to the 1,4-di­hydro­pyridine ring were refined isotropically for a stable structure. The atoms (C11/C11*A* and C12/C12*A*) of the dimethyl group attached to the cyclo­hexane ring, and the two carbon atoms (C7/C7*A* and C8/C8*A*) in the anti­clockwise direction after the carbon atom to which the dimethyl group of the cyclo­hexane ring is attached, were refined as disordered over two sets of sites in a 0.646 (3):0.354 (3) ratio.

## Supplementary Material

Crystal structure: contains datablock(s) I, II, III. DOI: 10.1107/S2056989024001233/jy2044sup1.cif

Structure factors: contains datablock(s) I. DOI: 10.1107/S2056989024001233/jy2044Isup2.hkl

Structure factors: contains datablock(s) II. DOI: 10.1107/S2056989024001233/jy2044IIsup3.hkl

Structure factors: contains datablock(s) III. DOI: 10.1107/S2056989024001233/jy2044IIIsup4.hkl

Supporting information file. DOI: 10.1107/S2056989024001233/jy2044Isup5.cml

Supporting information file. DOI: 10.1107/S2056989024001233/jy2044IIsup6.cml

Supporting information file. DOI: 10.1107/S2056989024001233/jy2044IIIsup7.cml

CCDC references: 2331194, 2331193, 2331192

Additional supporting information:  crystallographic information; 3D view; checkCIF report

## Figures and Tables

**Figure 6:**
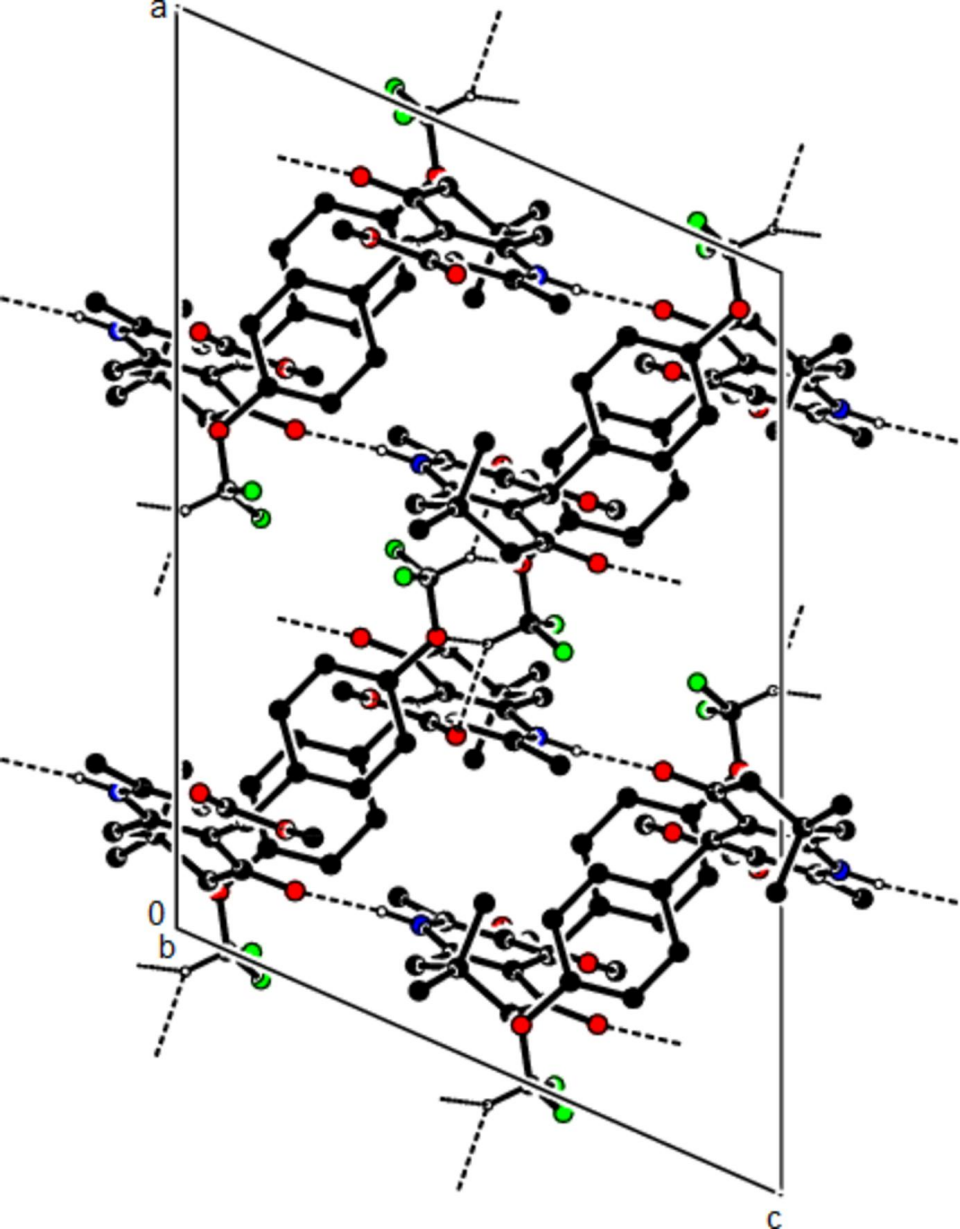
The N—H⋯O and C—H⋯O contacts (solid lines) of (**I**), shown along the *b*-axis.

**Figure 7:**
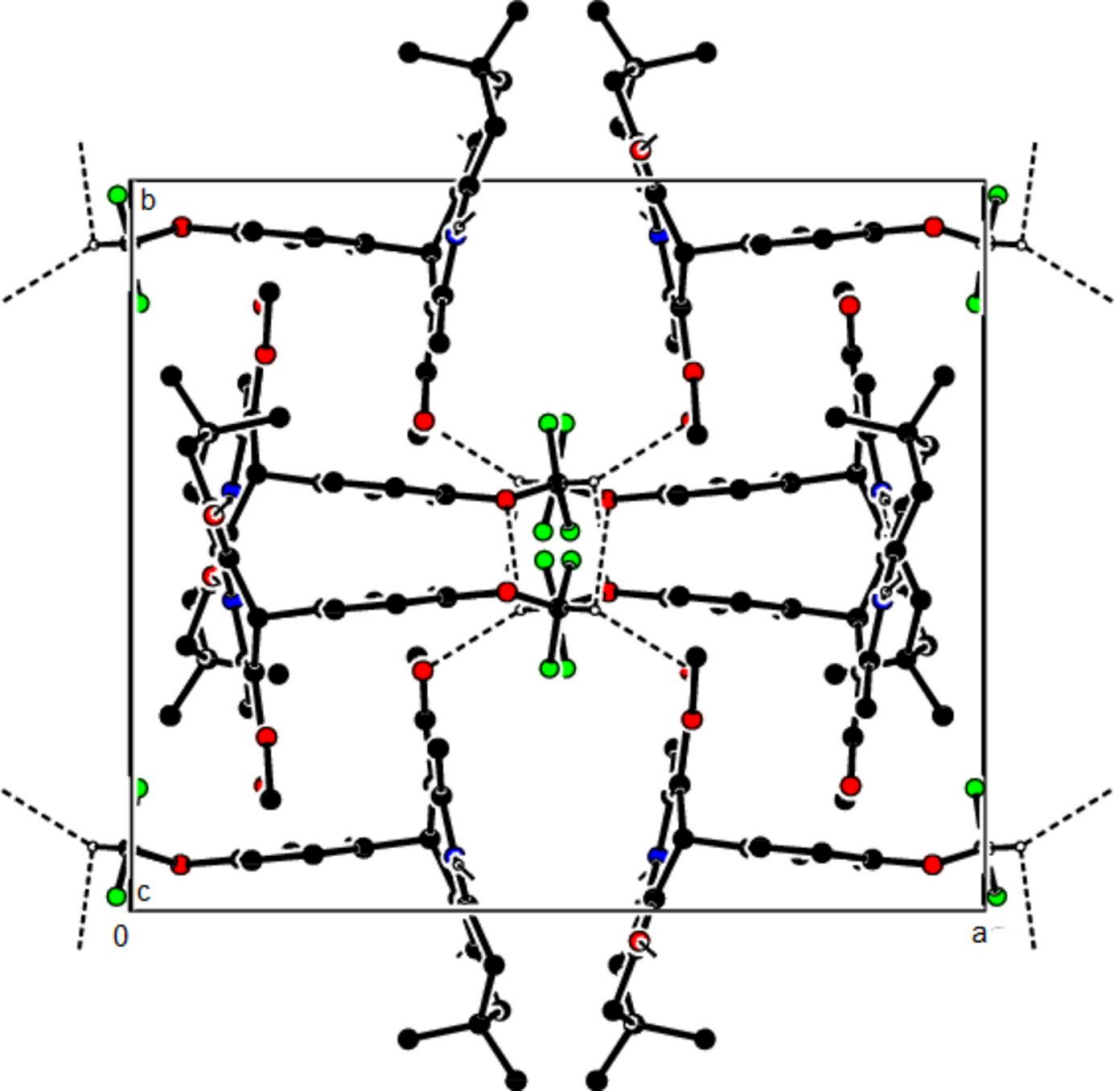
The N—H⋯O and C—H⋯O contacts (solid lines) of (**I**), shown along the *c*-axis.

**Figure 9:**
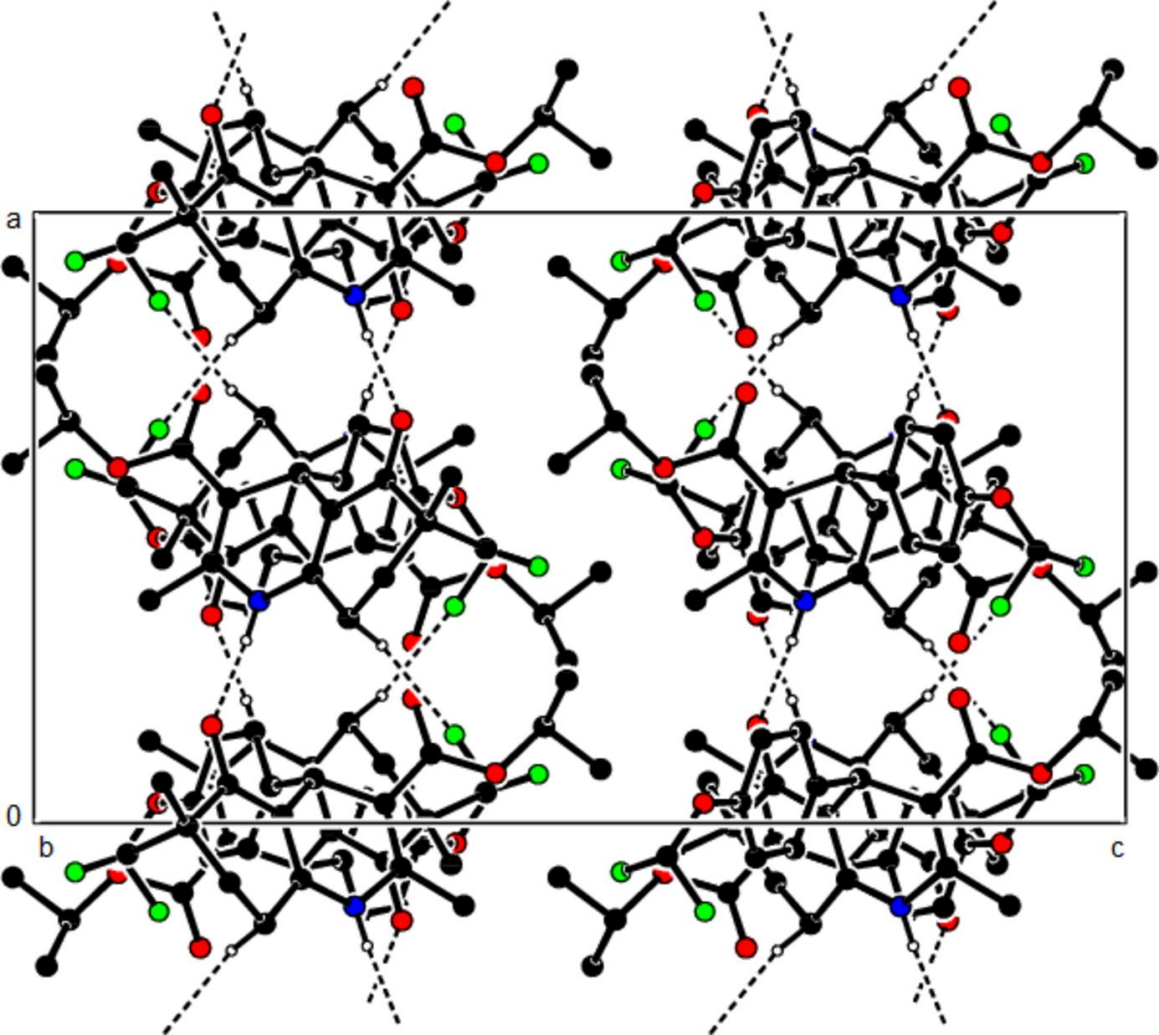
The N—H⋯O and C—H⋯F contacts (solid lines) of (**II**), shown along the *b*-axis.

**Figure 10:**
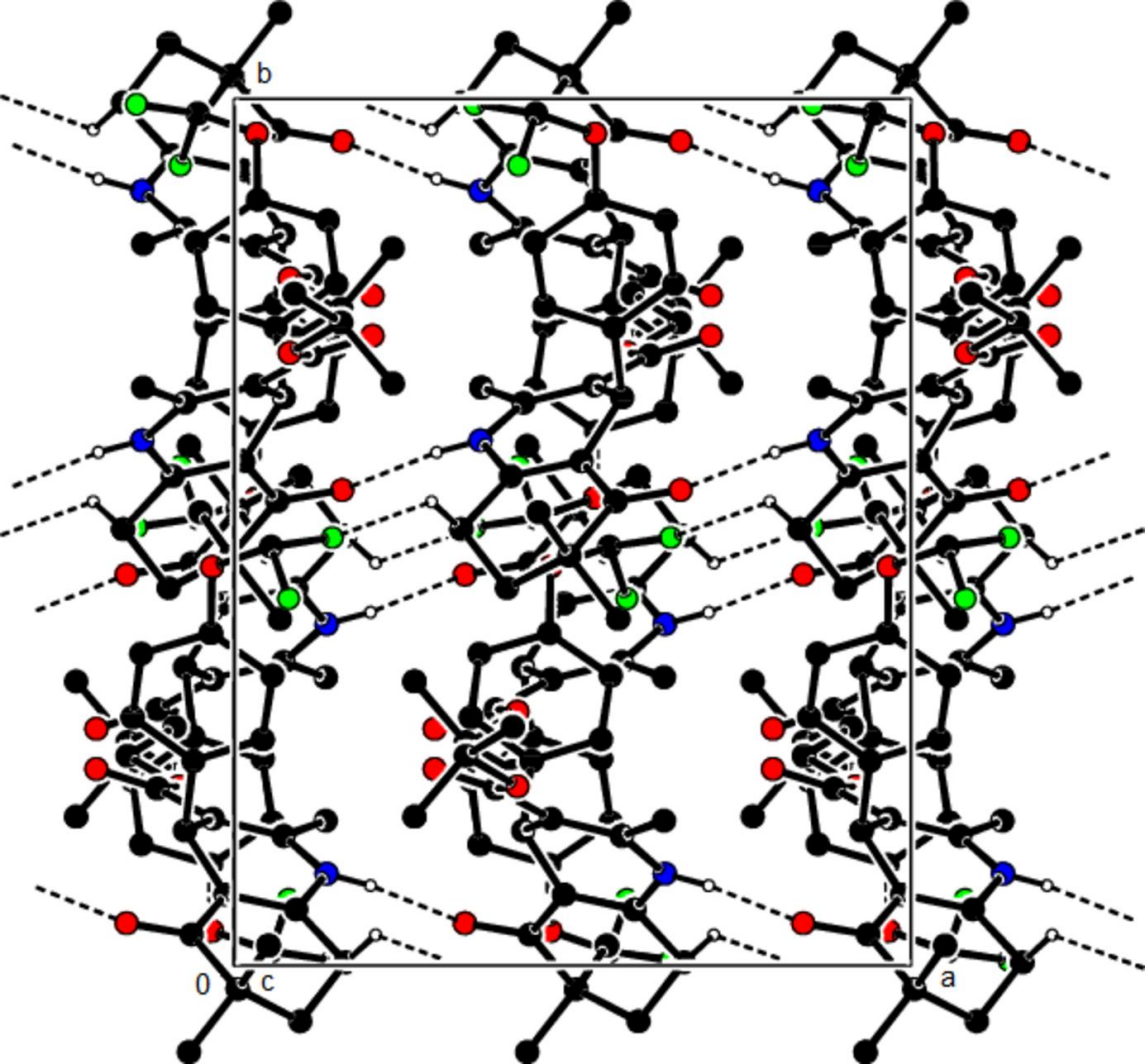
The N—H⋯O and C—H⋯F contacts (solid lines) of (**II**), shown along the *c*-axis.

**Figure 11:**
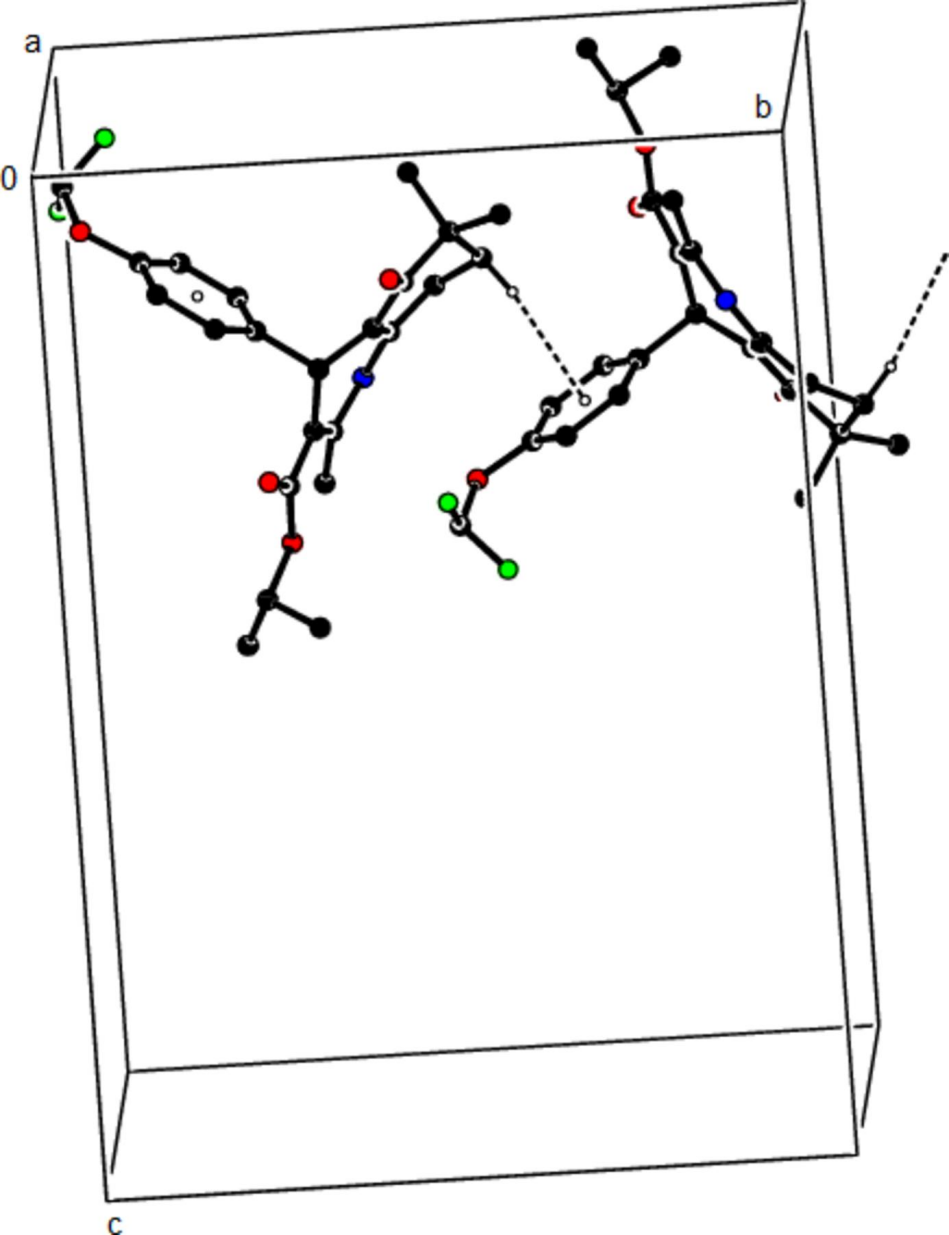
The C—H⋯π contacts (solid lines) of (**II**), shown along the *a*-axis.

**Figure 13:**
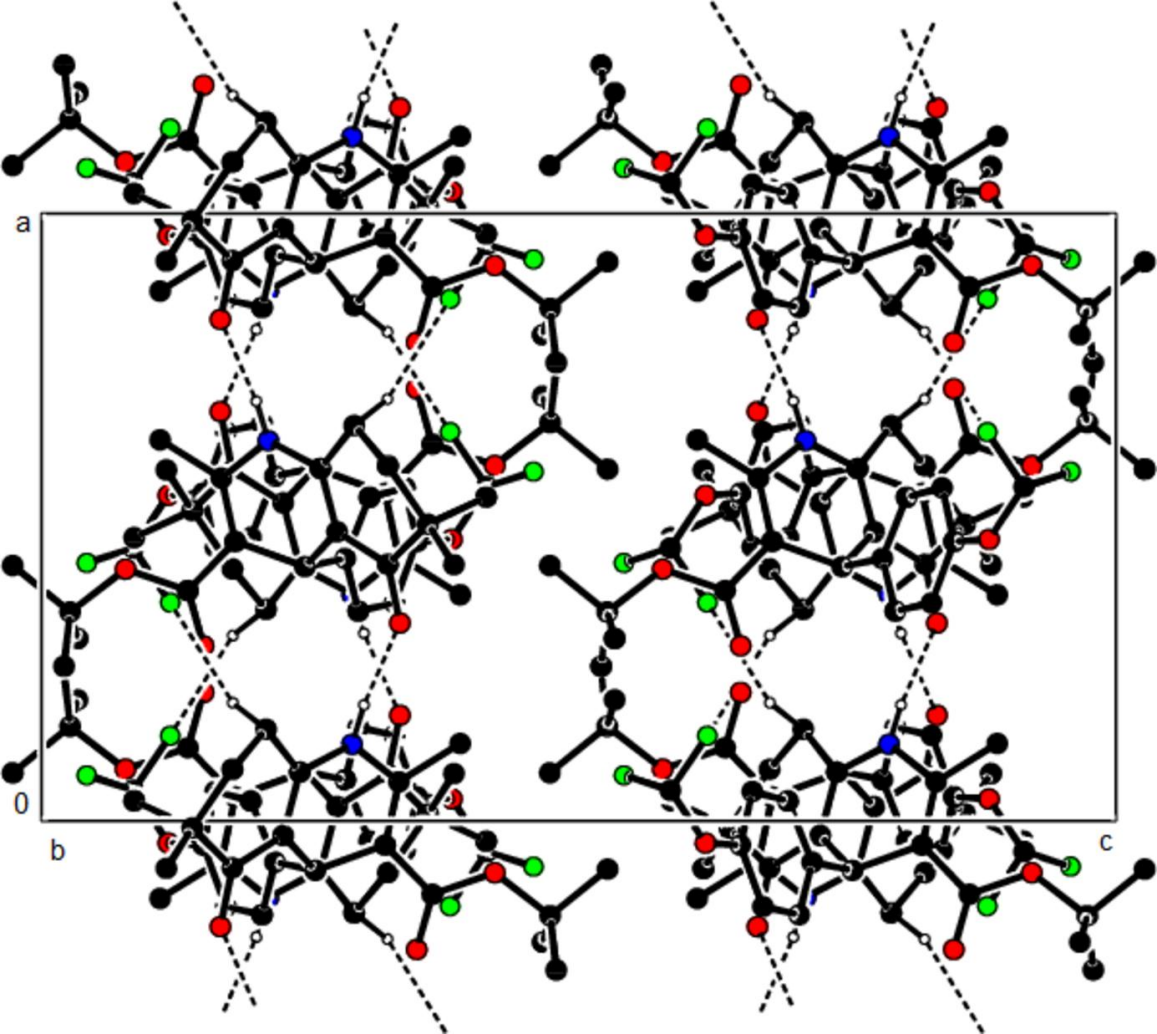
The N—H⋯O and C—H⋯F contacts (solid lines) of (**III**), shown along the *b*-axis.

**Figure 14:**
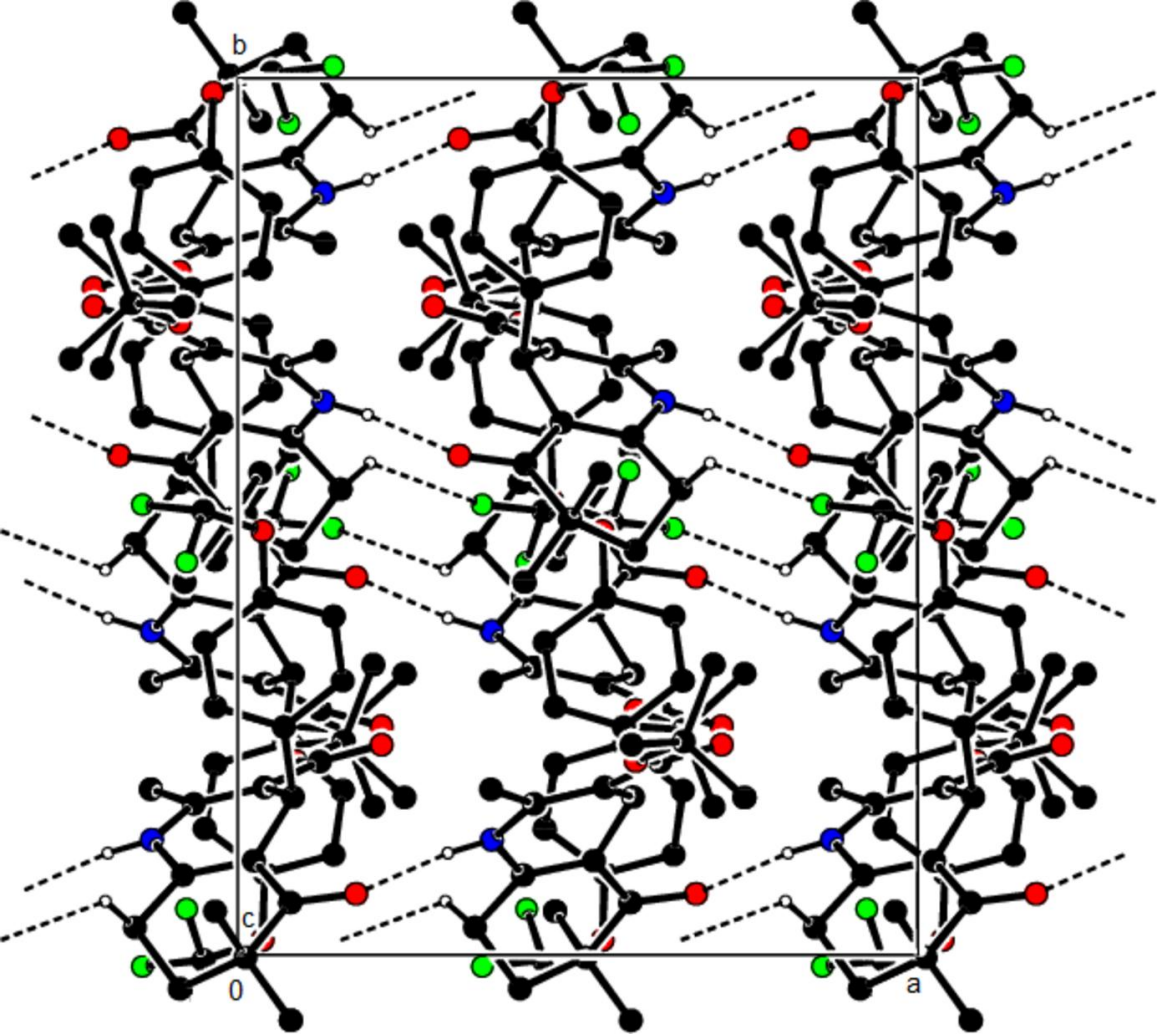
The N—H⋯O and C—H⋯F contacts (solid lines) of (**III**), shown along the *c*-axis.

**Figure 15:**
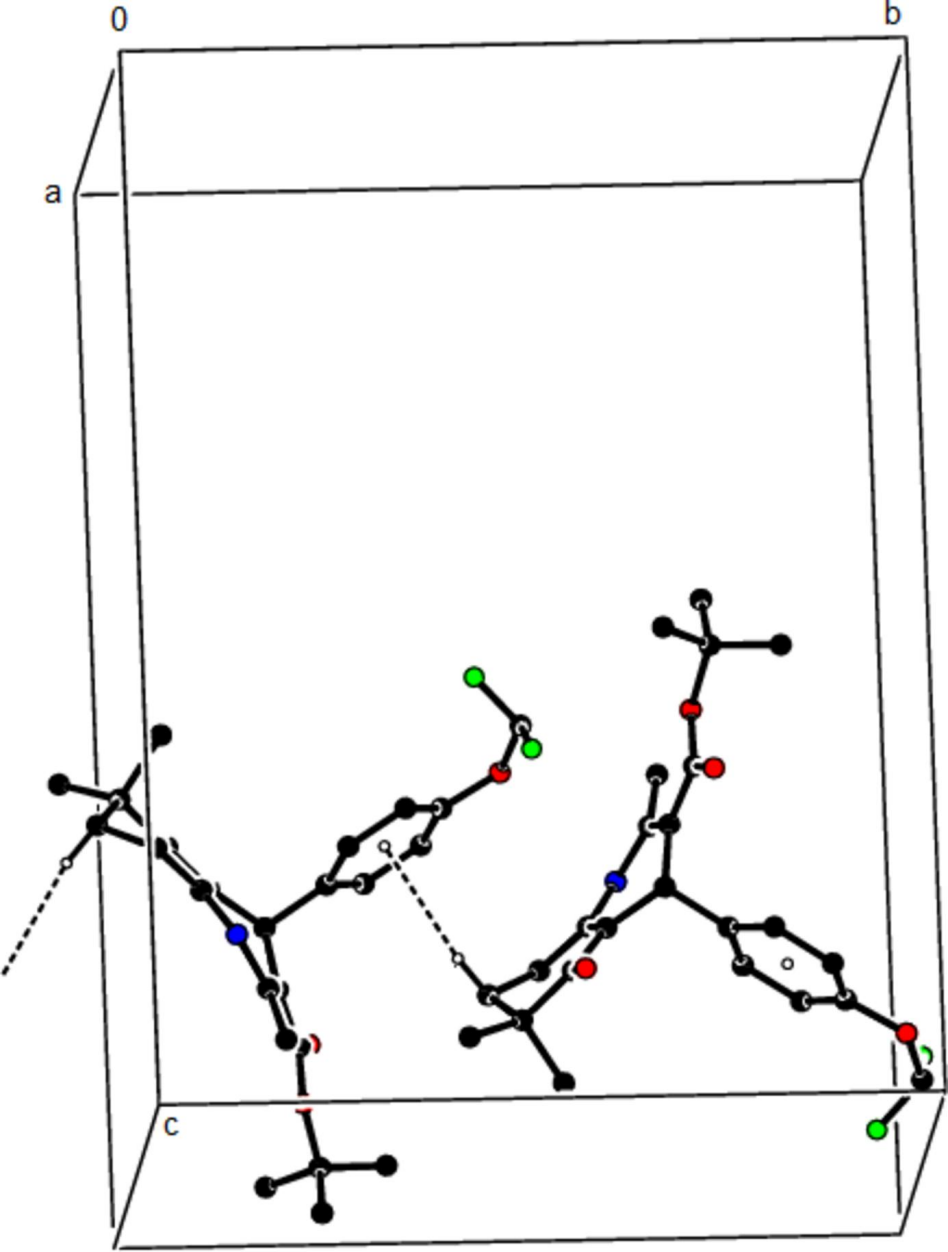
The C—H⋯π contacts (solid lines) of (**III**), shown along the *a*-axis.

**Table 1:** Hydrogen-bond geometry (Å, °) for (**I**)

*D*—H⋯*A*	*D*—H	H⋯*A*	*D*⋯*A*	*D*—H⋯*A*
N1—H1*N*⋯O1^i^	0.90 (3)	1.93 (4)	2.834 (3)	174 (3)
C12—H12*A*⋯O2	0.98	2.32	2.831 (4)	111
C12—H12*C*⋯F2^ii^	0.98	2.63	3.449 (5)	141
C12—H12*C*⋯F1*A* ^ii^	0.98	2.41	3.291 (7)	150
C14—H14*C*⋯O4*A* ^iii^	0.98	2.66	3.551 (6)	152
C17—H17*A*⋯F1	0.95	2.43	2.975 (4)	117
C17—H17*A*⋯F1^iv^	0.95	2.56	3.488 (4)	165
C21—H21*A*⋯O2^v^	1.00	2.44	3.155 (5)	128
C21*A*—H21*B*⋯O2^v^	1.00	2.50	3.062 (7)	115

Symmetry codes: (i) 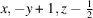 ; (ii) 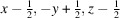 ; (iii) 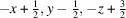 ; (iv) 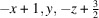 ; (v) 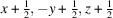 .

**Table 2:** Hydrogen-bond geometry (Å, °) for (**II**)

*D*—H⋯*A*	*D*—H	H⋯*A*	*D*⋯*A*	*D*—H⋯*A*
N1—H1*N*⋯O1^i^	0.863 (16)	1.967 (16)	2.8258 (12)	173.0 (14)
C2—H2*A*⋯F2^ii^	0.99	2.40	3.1626 (13)	133
C12—H12*A*⋯O3	0.98	2.18	2.7991 (14)	120
C19—H19*A*⋯F2	0.95	2.37	2.9106 (14)	116
C23—H23*A*⋯F1^iii^	1.00	2.63	3.3972 (14)	133

Symmetry codes: (i) 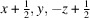 ; (ii) 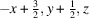 ; (iii) 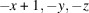 .

**Table 3:** Hydrogen-bond geometry (Å, °) for (**III**)

*D*—H⋯*A*	*D*—H	H⋯*A*	*D*⋯*A*	*D*—H⋯*A*
N1—H1*N*⋯O1^i^	0.88 (2)	1.97 (2)	2.8418 (16)	171.2 (19)
C8*A*—H8*A*⋯F2^ii^	0.99	2.53	3.168 (19)	130
C8*A*—H8*AB*⋯F2^ii^	0.99	2.48	3.168 (19)	126
C10—H10*A*⋯O4	0.98	2.27	2.7834 (18)	112
C15—H15*A*⋯O3	0.98	2.47	3.038 (3)	116
C16—H16*C*⋯F1^iii^	0.98	2.62	3.573 (2)	164
C17—H17*B*⋯O3	0.98	2.41	2.969 (2)	116
C22—H22*A*⋯F2	0.95	2.37	2.9091 (19)	116
C24—H24*A*⋯O4^iv^	1.00	2.65	3.4638 (18)	139

Symmetry codes: (i) 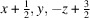 ; (ii) 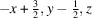 ; (iii) 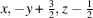 ; (iv) 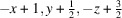 .

**Table 4:** Percentage contributions of inter­atomic contacts to the Hirshfeld surface for the compounds

Contact	Percentage contribution		
	(**I**)	(**II**)	(**III**)
H⋯H	49.1	55.5	58.9
O⋯H/H⋯O	17.5	14.9	12.7
F⋯H/H⋯F	16.2	14.1	12.9
C⋯H/H⋯C	11.7	14.5	12.0
F⋯F	1.8	–	0.2
O⋯C/C⋯O	–	1.2	1.0
F⋯O/O⋯F	0.8	–	0.2
N⋯H/H⋯N	0.5	0.2	0.2
F⋯C/C⋯F	0.5	1.5	1.4
O⋯N/N⋯O	0.3	0.5	0.4
O⋯O	0.1	–	–
C⋯C	0.1	0.4	0.1

**Table 5:** Experimental details

	(**I**)	(**II**)	(**III**)
Crystal data			
Chemical formula	C_21_H_23_F_2_NO_4_	C_23_H_27_F_2_NO_4_	C_24_H_29_F_2_NO_4_
*M*_r_	391.40	419.45	433.48
Crystal system, space group	Monoclinic, *C*2/*c*	Orthorhombic, *P* *b* *c* *a*	Orthorhombic, *P* *b* *c* *a*
Temperature (K)	100	100	100
*a*, *b*, *c* (Å)	19.705 (3), 15.389 (2), 14.1279 (19)	12.255 (3), 15.694 (3), 21.903 (4)	12.4094 (8), 15.9871 (12), 21.9629 (15)
α, β, γ (°)	90, 113.801 (4), 90	90, 90, 90	90, 90, 90
*V* (Å^3^)	3919.7 (9)	4212.3 (14)	4357.2 (5)
*Z*	8	8	8
Radiation type	Mo *K*α	Mo *K*α	Mo *K*α
μ (mm^−1^)	0.10	0.10	0.10
Crystal size (mm)	0.30 × 0.25 × 0.17	0.31 × 0.23 × 0.08	0.31 × 0.27 × 0.09

Data collection			
Diffractometer	Bruker D8 Quest with Photon 2 detector	Bruker D8 Quest with Photon 2 detector	Bruker APEXII CCD
Absorption correction	Multi-scan (*SADABS*; Krause *et al.*, 2015 ▸)	Multi-scan (*SADABS*; Krause *et al.*, 2015 ▸)	Multi-scan (*SADABS*; Krause *et al.*, 2015 ▸)
*T*_min_, *T*_max_	0.603, 0.746	0.684, 0.747	0.374, 0.746
No. of measured, independent and observed [*I* > 2σ(*I*)] reflections	47858, 4871, 3288	102650, 8537, 6743	56620, 6654, 4732
*R*_int_	0.082	0.073	0.142
(sin θ/λ)_max_ (Å^−1^)	0.667	0.788	0.715

Refinement			
*R*[*F* ^2^ > 2σ(*F* ^2^)], *wR*(*F* ^2^), *S*	0.073, 0.186, 1.05	0.050, 0.128, 1.03	0.059, 0.163, 1.05
No. of reflections	4871	8537	6654
No. of parameters	332	280	329
No. of restraints	361	0	68
H-atom treatment	H atoms treated by a mixture of independent and constrained refinement	H atoms treated by a mixture of independent and constrained refinement	H atoms treated by a mixture of independent and constrained refinement
Δρ_max_, Δρ_min_ (e Å^−3^)	0.58, −0.58	0.58, −0.42	0.37, −0.31

Computer programs: *APEX2* and *SAINT* (Bruker, 2018 ▸), *SHELXT* (Sheldrick, 2015*a* ▸), *SHELXL* (Sheldrick, 2015*b* ▸), *ORTEP-3 for Windows* (Farrugia, 2012 ▸) and *PLATON* (Spek, 2020 ▸).

## supplementary crystallographic information

### Methyl 4-[4-(difluoromethoxy)phenyl]-2,7,7-trimethyl-5-oxo-1,4,5,6,7,8-hexahydroquinoline-3-carboxylate (I) Crystal data

**Table:**

C_21_H_23_F_2_NO_4_	*F*(000) = 1648
*M**_r_* = 391.40	*D*_x_ = 1.326 Mg m^−^^3^
Monoclinic, *C*2/*c*	Mo *K*α radiation, λ = 0.71073 Å
*a* = 19.705 (3) Å	Cell parameters from 9994 reflections
*b* = 15.389 (2) Å	θ = 2.6–29.2°
*c* = 14.1279 (19) Å	µ = 0.10 mm^−^^1^
β = 113.801 (4)°	*T* = 100 K
*V* = 3919.7 (9) Å^3^	Prism, colorless
*Z* = 8	0.30 × 0.25 × 0.17 mm

### Methyl 4-[4-(difluoromethoxy)phenyl]-2,7,7-trimethyl-5-oxo-1,4,5,6,7,8-hexahydroquinoline-3-carboxylate (I) Data collection

**Table:**

Bruker D8 Quest with Photon 2 detector diffractometer	3288 reflections with *I* > 2σ(*I*)
φ and ω scans	*R*_int_ = 0.082
Absorption correction: multi-scan (SADABS; Krause *et al.*, 2015)	θ_max_ = 28.3°, θ_min_ = 1.7°
*T*_min_ = 0.603, *T*_max_ = 0.746	*h* = −26→24
47858 measured reflections	*k* = −20→20
4871 independent reflections	*l* = −18→18

### Methyl 4-[4-(difluoromethoxy)phenyl]-2,7,7-trimethyl-5-oxo-1,4,5,6,7,8-hexahydroquinoline-3-carboxylate (I) Refinement

**Table:**

Refinement on *F*^2^	Primary atom site location: dual
Least-squares matrix: full	Secondary atom site location: difference Fourier map
*R*[*F*^2^ > 2σ(*F*^2^)] = 0.073	Hydrogen site location: mixed
*wR*(*F*^2^) = 0.186	H atoms treated by a mixture of independent and constrained refinement
*S* = 1.05	*w* = 1/[σ^2^(*F*_o_^2^) + (0.043*P*)^2^ + 11.081*P*] where *P* = (*F*_o_^2^ + 2*F*_c_^2^)/3
4871 reflections	(Δ/σ)_max_ = 0.001
332 parameters	Δρ_max_ = 0.58 e Å^−^^3^
361 restraints	Δρ_min_ = −0.58 e Å^−^^3^

### Methyl 4-[4-(difluoromethoxy)phenyl]-2,7,7-trimethyl-5-oxo-1,4,5,6,7,8-hexahydroquinoline-3-carboxylate (I) Special details

**Table:**

Geometry. All esds (except the esd in the dihedral angle between two l.s. planes) are estimated using the full covariance matrix. The cell esds are taken into account individually in the estimation of esds in distances, angles and torsion angles; correlations between esds in cell parameters are only used when they are defined by crystal symmetry. An approximate (isotropic) treatment of cell esds is used for estimating esds involving l.s. planes.

### Methyl 4-[4-(difluoromethoxy)phenyl]-2,7,7-trimethyl-5-oxo-1,4,5,6,7,8-hexahydroquinoline-3-carboxylate (I) Fractional atomic coordinates and isotropic or equivalent isotropic displacement parameters (Å^2^)

**Table:**

	*x*	*y*	*z*	*U*_iso_*/*U*_eq_	Occ. (<1)
O1	0.09716 (12)	0.54160 (14)	0.69583 (14)	0.0510 (5)	
O2	0.15731 (12)	0.17128 (13)	0.53819 (18)	0.0594 (6)	
O3	0.15902 (12)	0.23777 (13)	0.68037 (16)	0.0553 (6)	
N1	0.12023 (13)	0.42587 (15)	0.40402 (16)	0.0392 (5)	
C1	0.10494 (13)	0.49275 (16)	0.45557 (17)	0.0320 (5)	
C2	0.07419 (16)	0.57420 (17)	0.39524 (18)	0.0432 (7)	
H2A	0.019785	0.568190	0.357785	0.052*	
H2B	0.095506	0.581955	0.343212	0.052*	
C3	0.09123 (15)	0.65493 (18)	0.4646 (2)	0.0432 (7)	
C4	0.06672 (14)	0.63670 (17)	0.5521 (2)	0.0392 (6)	
H4A	0.084333	0.684614	0.603066	0.047*	
H4B	0.011840	0.636679	0.523487	0.047*	
C5	0.09443 (13)	0.55203 (17)	0.60759 (18)	0.0336 (5)	
C6	0.11516 (12)	0.48321 (15)	0.55589 (16)	0.0292 (5)	
C7	0.14879 (14)	0.40124 (16)	0.61533 (18)	0.0332 (5)	
C8	0.14482 (13)	0.32712 (16)	0.54191 (19)	0.0342 (5)	
C9	0.13559 (14)	0.34270 (17)	0.4431 (2)	0.0377 (6)	
C10	0.0482 (2)	0.7326 (2)	0.4006 (3)	0.0720 (12)	
H10A	−0.005015	0.719741	0.371558	0.108*	
H10B	0.064070	0.743656	0.344258	0.108*	
H10C	0.058084	0.784075	0.444977	0.108*	
C11	0.17443 (16)	0.67588 (19)	0.5086 (2)	0.0457 (7)	
H11A	0.184624	0.725991	0.555128	0.068*	
H11B	0.189114	0.689477	0.451748	0.068*	
H11C	0.202669	0.625559	0.547039	0.068*	
C12	0.13988 (19)	0.2777 (2)	0.3665 (2)	0.0547 (8)	
H12A	0.177848	0.234171	0.402577	0.082*	
H12B	0.152879	0.307481	0.314772	0.082*	
H12C	0.091700	0.248966	0.332042	0.082*	
C13	0.15455 (14)	0.23808 (18)	0.5819 (2)	0.0429 (6)	
C14	0.1635 (2)	0.1524 (2)	0.7254 (3)	0.0746 (11)	
H14A	0.167604	0.158165	0.796548	0.112*	
H14B	0.207276	0.121931	0.725548	0.112*	
H14C	0.118808	0.119180	0.684523	0.112*	
C15	0.22622 (13)	0.4139 (4)	0.6988 (2)	0.0329 (10)	0.647 (3)
C16	0.28551 (17)	0.4229 (3)	0.67009 (18)	0.0388 (11)	0.647 (3)
H16A	0.276995	0.423772	0.598944	0.047*	0.647 (3)
C17	0.35727 (14)	0.4308 (2)	0.7454 (3)	0.0430 (10)	0.647 (3)
H17A	0.397791	0.436936	0.725802	0.052*	0.647 (3)
C18	0.36973 (14)	0.4295 (2)	0.8495 (2)	0.0426 (11)	0.647 (3)
C19	0.31044 (19)	0.4205 (3)	0.87829 (18)	0.0449 (11)	0.647 (3)
H19A	0.318964	0.419649	0.949438	0.054*	0.647 (3)
C20	0.23869 (16)	0.4127 (3)	0.8029 (3)	0.0414 (11)	0.647 (3)
H20A	0.198167	0.406484	0.822581	0.050*	0.647 (3)
O4	0.44015 (17)	0.4369 (2)	0.9302 (2)	0.0575 (10)	0.647 (3)
C21	0.5006 (3)	0.4146 (5)	0.9161 (5)	0.081 (2)	0.647 (3)
H21A	0.542852	0.412128	0.985748	0.097*	0.647 (3)
F1	0.51546 (16)	0.4803 (3)	0.8608 (3)	0.0920 (13)	0.647 (3)
F2	0.4900 (2)	0.3321 (2)	0.8754 (4)	0.0751 (13)	0.647 (3)
C15A	0.2335 (2)	0.4232 (7)	0.6827 (5)	0.0348 (18)	0.353 (3)
C16A	0.2824 (3)	0.4423 (5)	0.6369 (4)	0.0375 (18)	0.353 (3)
H16B	0.264737	0.445770	0.563751	0.045*	0.353 (3)
C17A	0.3570 (3)	0.4564 (4)	0.6983 (4)	0.0342 (15)	0.353 (3)
H17B	0.390378	0.469487	0.667015	0.041*	0.353 (3)
C18A	0.3828 (2)	0.4514 (4)	0.8053 (4)	0.0373 (15)	0.353 (3)
C19A	0.3339 (3)	0.4323 (5)	0.8511 (4)	0.0391 (16)	0.353 (3)
H19B	0.351570	0.428885	0.924261	0.047*	0.353 (3)
C20A	0.2593 (3)	0.4182 (6)	0.7897 (5)	0.0387 (17)	0.353 (3)
H20B	0.225928	0.405167	0.820999	0.046*	0.353 (3)
O4A	0.4570 (2)	0.4712 (3)	0.8666 (4)	0.0458 (14)	0.353 (3)
C21A	0.5040 (4)	0.4078 (6)	0.9164 (6)	0.068 (3)	0.353 (3)
H21B	0.554500	0.433982	0.951241	0.081*	0.353 (3)
F1A	0.5034 (3)	0.3581 (5)	0.8348 (4)	0.0512 (16)	0.353 (3)
F2A	0.4849 (3)	0.3731 (4)	0.9915 (4)	0.0766 (19)	0.353 (3)
H1N	0.1163 (19)	0.437 (2)	0.339 (3)	0.062 (10)*	
H7	0.1208 (15)	0.3857 (17)	0.655 (2)	0.032 (7)*	

### Methyl 4-[4-(difluoromethoxy)phenyl]-2,7,7-trimethyl-5-oxo-1,4,5,6,7,8-hexahydroquinoline-3-carboxylate (I) Atomic displacement parameters (Å^2^)

**Table:**

	*U*^11^	*U*^22^	*U*^33^	*U*^12^	*U*^13^	*U*^23^
O1	0.0691 (14)	0.0580 (13)	0.0330 (10)	0.0189 (10)	0.0279 (10)	0.0037 (9)
O2	0.0485 (12)	0.0372 (11)	0.0726 (15)	−0.0057 (9)	0.0037 (11)	−0.0120 (10)
O3	0.0576 (13)	0.0419 (11)	0.0525 (12)	0.0102 (9)	0.0079 (10)	0.0168 (9)
N1	0.0505 (13)	0.0445 (12)	0.0249 (10)	−0.0165 (10)	0.0177 (9)	−0.0080 (9)
C1	0.0326 (12)	0.0362 (12)	0.0232 (11)	−0.0098 (10)	0.0071 (9)	−0.0013 (9)
C2	0.0513 (16)	0.0430 (14)	0.0220 (11)	−0.0171 (12)	0.0008 (11)	0.0029 (10)
C3	0.0431 (15)	0.0394 (14)	0.0312 (13)	−0.0079 (11)	−0.0017 (11)	0.0056 (10)
C4	0.0329 (13)	0.0366 (13)	0.0387 (13)	0.0039 (10)	0.0047 (10)	0.0011 (11)
C5	0.0293 (12)	0.0429 (14)	0.0268 (11)	0.0042 (10)	0.0094 (9)	0.0015 (10)
C6	0.0266 (11)	0.0351 (12)	0.0223 (10)	0.0006 (9)	0.0061 (9)	0.0006 (9)
C7	0.0314 (12)	0.0382 (13)	0.0266 (11)	0.0062 (10)	0.0083 (10)	0.0019 (10)
C8	0.0256 (11)	0.0342 (12)	0.0382 (13)	−0.0008 (9)	0.0081 (10)	−0.0040 (10)
C9	0.0348 (13)	0.0401 (14)	0.0387 (13)	−0.0116 (11)	0.0154 (11)	−0.0121 (11)
C10	0.072 (2)	0.0413 (17)	0.062 (2)	−0.0130 (15)	−0.0159 (17)	0.0200 (15)
C11	0.0473 (16)	0.0479 (16)	0.0353 (13)	−0.0163 (13)	0.0101 (12)	−0.0057 (12)
C12	0.0607 (19)	0.0528 (18)	0.0557 (18)	−0.0183 (15)	0.0288 (15)	−0.0275 (15)
C13	0.0257 (12)	0.0411 (15)	0.0479 (15)	−0.0017 (10)	0.0002 (11)	0.0012 (12)
C14	0.070 (2)	0.051 (2)	0.080 (2)	0.0077 (17)	0.0066 (19)	0.0312 (18)
C15	0.0309 (19)	0.034 (2)	0.028 (2)	0.0096 (16)	0.0063 (15)	0.0006 (17)
C16	0.0326 (19)	0.050 (3)	0.0262 (19)	0.0093 (18)	0.0040 (16)	−0.0102 (19)
C17	0.0315 (19)	0.053 (2)	0.034 (2)	0.0103 (17)	0.0024 (17)	−0.0150 (19)
C18	0.040 (2)	0.037 (2)	0.0295 (19)	0.0117 (18)	−0.0084 (18)	−0.0116 (16)
C19	0.059 (3)	0.043 (2)	0.0213 (18)	0.013 (2)	0.0046 (17)	−0.0017 (16)
C20	0.047 (2)	0.045 (2)	0.0258 (18)	0.013 (2)	0.0087 (17)	0.0010 (16)
O4	0.0514 (17)	0.0443 (17)	0.0397 (16)	0.0138 (14)	−0.0202 (13)	−0.0107 (13)
C21	0.047 (3)	0.075 (3)	0.076 (3)	0.025 (3)	−0.021 (3)	−0.036 (3)
F1	0.0350 (16)	0.141 (3)	0.077 (2)	0.0084 (18)	−0.0005 (15)	−0.059 (2)
F2	0.0411 (19)	0.065 (2)	0.093 (3)	0.0140 (15)	−0.0006 (19)	−0.028 (2)
C15A	0.039 (3)	0.034 (3)	0.022 (3)	0.007 (3)	0.002 (3)	−0.005 (3)
C16A	0.031 (3)	0.037 (4)	0.034 (3)	0.004 (3)	0.002 (3)	−0.002 (3)
C17A	0.032 (3)	0.032 (3)	0.032 (3)	0.001 (2)	0.006 (3)	−0.005 (3)
C18A	0.033 (3)	0.036 (3)	0.031 (3)	0.004 (2)	0.001 (2)	−0.004 (3)
C19A	0.035 (3)	0.043 (3)	0.027 (3)	−0.003 (3)	−0.001 (3)	0.000 (3)
C20A	0.035 (3)	0.044 (3)	0.031 (3)	0.001 (3)	0.007 (3)	−0.002 (3)
O4A	0.034 (2)	0.041 (3)	0.043 (3)	0.002 (2)	−0.004 (2)	0.004 (2)
C21A	0.045 (4)	0.060 (4)	0.065 (5)	0.013 (4)	−0.012 (4)	−0.011 (4)
F1A	0.033 (3)	0.080 (4)	0.038 (3)	0.013 (3)	0.011 (2)	−0.020 (3)
F2A	0.055 (3)	0.110 (5)	0.062 (3)	0.005 (3)	0.021 (3)	0.046 (3)

### Methyl 4-[4-(difluoromethoxy)phenyl]-2,7,7-trimethyl-5-oxo-1,4,5,6,7,8-hexahydroquinoline-3-carboxylate (I) Geometric parameters (Å, º)

**Table:**

O1—C5	1.236 (3)	C12—H12C	0.9800
O2—C13	1.212 (3)	C14—H14A	0.9800
O3—C13	1.358 (4)	C14—H14B	0.9800
O3—C14	1.447 (4)	C14—H14C	0.9800
N1—C1	1.363 (3)	C15—C16	1.3900
N1—C9	1.378 (3)	C15—C20	1.3900
N1—H1N	0.90 (3)	C16—C17	1.3900
C1—C6	1.357 (3)	C16—H16A	0.9500
C1—C2	1.499 (3)	C17—C18	1.3900
C2—C3	1.533 (4)	C17—H17A	0.9500
C2—H2A	0.9900	C18—C19	1.3900
C2—H2B	0.9900	C18—O4	1.400 (3)
C3—C4	1.524 (4)	C19—C20	1.3900
C3—C10	1.532 (4)	C19—H19A	0.9500
C3—C11	1.535 (4)	C20—H20A	0.9500
C4—C5	1.505 (3)	O4—C21	1.330 (6)
C4—H4A	0.9900	C21—F2	1.375 (7)
C4—H4B	0.9900	C21—F1	1.379 (8)
C5—C6	1.436 (3)	C21—H21A	1.0000
C6—C7	1.512 (3)	C15A—C16A	1.3900
C7—C15	1.518 (3)	C15A—C20A	1.3900
C7—C8	1.522 (3)	C16A—C17A	1.3900
C7—C15A	1.589 (4)	C16A—H16B	0.9500
C7—H7	0.97 (3)	C17A—C18A	1.3900
C8—C9	1.354 (4)	C17A—H17B	0.9500
C8—C13	1.465 (4)	C18A—C19A	1.3900
C9—C12	1.501 (4)	C18A—O4A	1.399 (4)
C10—H10A	0.9800	C19A—C20A	1.3900
C10—H10B	0.9800	C19A—H19B	0.9500
C10—H10C	0.9800	C20A—H20B	0.9500
C11—H11A	0.9800	O4A—C21A	1.334 (7)
C11—H11B	0.9800	C21A—F2A	1.370 (7)
C11—H11C	0.9800	C21A—F1A	1.379 (9)
C12—H12A	0.9800	C21A—H21B	1.0000
C12—H12B	0.9800		
			
C13—O3—C14	114.9 (3)	H12A—C12—H12C	109.5
C1—N1—C9	123.2 (2)	H12B—C12—H12C	109.5
C1—N1—H1N	117 (2)	O2—C13—O3	121.4 (3)
C9—N1—H1N	119 (2)	O2—C13—C8	128.5 (3)
C6—C1—N1	120.3 (2)	O3—C13—C8	110.0 (2)
C6—C1—C2	122.7 (2)	O3—C14—H14A	109.5
N1—C1—C2	116.9 (2)	O3—C14—H14B	109.5
C1—C2—C3	112.34 (19)	H14A—C14—H14B	109.5
C1—C2—H2A	109.1	O3—C14—H14C	109.5
C3—C2—H2A	109.1	H14A—C14—H14C	109.5
C1—C2—H2B	109.1	H14B—C14—H14C	109.5
C3—C2—H2B	109.1	C16—C15—C20	120.0
H2A—C2—H2B	107.9	C16—C15—C7	119.0 (2)
C4—C3—C10	110.0 (3)	C20—C15—C7	120.9 (2)
C4—C3—C2	108.1 (2)	C15—C16—C17	120.0
C10—C3—C2	109.2 (2)	C15—C16—H16A	120.0
C4—C3—C11	110.3 (2)	C17—C16—H16A	120.0
C10—C3—C11	108.7 (2)	C16—C17—C18	120.0
C2—C3—C11	110.6 (3)	C16—C17—H17A	120.0
C5—C4—C3	114.5 (2)	C18—C17—H17A	120.0
C5—C4—H4A	108.6	C19—C18—C17	120.0
C3—C4—H4A	108.6	C19—C18—O4	116.4 (3)
C5—C4—H4B	108.6	C17—C18—O4	123.6 (3)
C3—C4—H4B	108.6	C18—C19—C20	120.0
H4A—C4—H4B	107.6	C18—C19—H19A	120.0
O1—C5—C6	120.8 (2)	C20—C19—H19A	120.0
O1—C5—C4	119.8 (2)	C19—C20—C15	120.0
C6—C5—C4	119.5 (2)	C19—C20—H20A	120.0
C1—C6—C5	119.9 (2)	C15—C20—H20A	120.0
C1—C6—C7	121.2 (2)	C21—O4—C18	120.7 (3)
C5—C6—C7	118.9 (2)	O4—C21—F2	107.8 (6)
C6—C7—C15	113.7 (3)	O4—C21—F1	107.6 (5)
C6—C7—C8	110.86 (19)	F2—C21—F1	118.1 (5)
C15—C7—C8	112.6 (3)	O4—C21—H21A	107.6
C6—C7—C15A	105.9 (4)	F2—C21—H21A	107.6
C8—C7—C15A	108.7 (4)	F1—C21—H21A	107.6
C6—C7—H7	107.8 (15)	C16A—C15A—C20A	120.0
C15—C7—H7	102.1 (15)	C16A—C15A—C7	121.6 (4)
C8—C7—H7	109.2 (15)	C20A—C15A—C7	118.3 (4)
C15A—C7—H7	114.3 (16)	C17A—C16A—C15A	120.0
C9—C8—C13	120.4 (2)	C17A—C16A—H16B	120.0
C9—C8—C7	121.2 (2)	C15A—C16A—H16B	120.0
C13—C8—C7	118.4 (2)	C16A—C17A—C18A	120.0
C8—C9—N1	119.6 (2)	C16A—C17A—H17B	120.0
C8—C9—C12	127.0 (3)	C18A—C17A—H17B	120.0
N1—C9—C12	113.4 (2)	C19A—C18A—C17A	120.0
C3—C10—H10A	109.5	C19A—C18A—O4A	120.3 (4)
C3—C10—H10B	109.5	C17A—C18A—O4A	119.5 (4)
H10A—C10—H10B	109.5	C18A—C19A—C20A	120.0
C3—C10—H10C	109.5	C18A—C19A—H19B	120.0
H10A—C10—H10C	109.5	C20A—C19A—H19B	120.0
H10B—C10—H10C	109.5	C19A—C20A—C15A	120.0
C3—C11—H11A	109.5	C19A—C20A—H20B	120.0
C3—C11—H11B	109.5	C15A—C20A—H20B	120.0
H11A—C11—H11B	109.5	C21A—O4A—C18A	119.8 (5)
C3—C11—H11C	109.5	O4A—C21A—F2A	110.1 (7)
H11A—C11—H11C	109.5	O4A—C21A—F1A	101.2 (6)
H11B—C11—H11C	109.5	F2A—C21A—F1A	121.3 (7)
C9—C12—H12A	109.5	O4A—C21A—H21B	107.8
C9—C12—H12B	109.5	F2A—C21A—H21B	107.8
H12A—C12—H12B	109.5	F1A—C21A—H21B	107.8
C9—C12—H12C	109.5		
			
C9—N1—C1—C6	10.0 (4)	C9—C8—C13—O2	1.7 (4)
C9—N1—C1—C2	−167.7 (2)	C7—C8—C13—O2	−175.6 (3)
C6—C1—C2—C3	28.0 (4)	C9—C8—C13—O3	−177.0 (2)
N1—C1—C2—C3	−154.2 (2)	C7—C8—C13—O3	5.7 (3)
C1—C2—C3—C4	−51.7 (3)	C6—C7—C15—C16	−74.8 (3)
C1—C2—C3—C10	−171.3 (3)	C8—C7—C15—C16	52.4 (4)
C1—C2—C3—C11	69.1 (3)	C6—C7—C15—C20	108.0 (3)
C10—C3—C4—C5	169.0 (2)	C8—C7—C15—C20	−124.8 (3)
C2—C3—C4—C5	49.9 (3)	C20—C15—C16—C17	0.0
C11—C3—C4—C5	−71.1 (3)	C7—C15—C16—C17	−177.2 (4)
C3—C4—C5—O1	158.9 (2)	C15—C16—C17—C18	0.0
C3—C4—C5—C6	−23.1 (3)	C16—C17—C18—C19	0.0
N1—C1—C6—C5	−176.0 (2)	C16—C17—C18—O4	179.9 (4)
C2—C1—C6—C5	1.6 (4)	C17—C18—C19—C20	0.0
N1—C1—C6—C7	4.5 (4)	O4—C18—C19—C20	−179.9 (3)
C2—C1—C6—C7	−177.9 (2)	C18—C19—C20—C15	0.0
O1—C5—C6—C1	173.5 (2)	C16—C15—C20—C19	0.0
C4—C5—C6—C1	−4.5 (3)	C7—C15—C20—C19	177.1 (4)
O1—C5—C6—C7	−7.0 (4)	C19—C18—O4—C21	155.4 (5)
C4—C5—C6—C7	175.0 (2)	C17—C18—O4—C21	−24.5 (6)
C1—C6—C7—C15	110.1 (3)	C18—O4—C21—F2	−51.8 (7)
C5—C6—C7—C15	−69.4 (3)	C18—O4—C21—F1	76.6 (6)
C1—C6—C7—C8	−18.1 (3)	C6—C7—C15A—C16A	−64.9 (6)
C5—C6—C7—C8	162.4 (2)	C8—C7—C15A—C16A	54.3 (6)
C1—C6—C7—C15A	99.6 (4)	C6—C7—C15A—C20A	118.7 (4)
C5—C6—C7—C15A	−79.9 (4)	C8—C7—C15A—C20A	−122.1 (4)
C6—C7—C8—C9	20.0 (3)	C20A—C15A—C16A—C17A	0.0
C15—C7—C8—C9	−108.7 (3)	C7—C15A—C16A—C17A	−176.4 (8)
C15A—C7—C8—C9	−96.0 (4)	C15A—C16A—C17A—C18A	0.0
C6—C7—C8—C13	−162.7 (2)	C16A—C17A—C18A—C19A	0.0
C15—C7—C8—C13	68.6 (3)	C16A—C17A—C18A—O4A	−175.9 (6)
C15A—C7—C8—C13	81.3 (4)	C17A—C18A—C19A—C20A	0.0
C13—C8—C9—N1	174.5 (2)	O4A—C18A—C19A—C20A	175.9 (6)
C7—C8—C9—N1	−8.2 (4)	C18A—C19A—C20A—C15A	0.0
C13—C8—C9—C12	−5.2 (4)	C16A—C15A—C20A—C19A	0.0
C7—C8—C9—C12	172.0 (2)	C7—C15A—C20A—C19A	176.5 (8)
C1—N1—C9—C8	−8.1 (4)	C19A—C18A—O4A—C21A	75.5 (8)
C1—N1—C9—C12	171.7 (2)	C17A—C18A—O4A—C21A	−108.6 (7)
C14—O3—C13—O2	−2.9 (4)	C18A—O4A—C21A—F2A	−66.7 (9)
C14—O3—C13—C8	175.9 (2)	C18A—O4A—C21A—F1A	62.8 (8)

### Methyl 4-[4-(difluoromethoxy)phenyl]-2,7,7-trimethyl-5-oxo-1,4,5,6,7,8-hexahydroquinoline-3-carboxylate (I) Hydrogen-bond geometry (Å, º)

**Table:**

*D*—H···*A*	*D*—H	H···*A*	*D*···*A*	*D*—H···*A*
N1—H1*N*···O1^i^	0.90 (3)	1.93 (4)	2.834 (3)	174 (3)
C12—H12*A*···O2	0.98	2.32	2.831 (4)	111
C12—H12*C*···F2^ii^	0.98	2.63	3.449 (5)	141
C12—H12*C*···F1*A*^ii^	0.98	2.41	3.291 (7)	150
C14—H14*C*···O4*A*^iii^	0.98	2.66	3.551 (6)	152
C17—H17*A*···F1	0.95	2.43	2.975 (4)	117
C17—H17*A*···F1^iv^	0.95	2.56	3.488 (4)	165
C21—H21*A*···O2^v^	1.00	2.44	3.155 (5)	128
C21*A*—H21*B*···O2^v^	1.00	2.50	3.062 (7)	115

Symmetry codes: (i) *x*, −*y*+1, *z*−1/2; (ii) *x*−1/2, −*y*+1/2, *z*−1/2; (iii) −*x*+1/2, *y*−1/2, −*z*+3/2; (iv) −*x*+1, *y*, −*z*+3/2; (v) *x*+1/2, −*y*+1/2, *z*+1/2.

### Isopropyl 4-[4-(difluoromethoxy)phenyl]-2,6,6-trimethyl-5-oxo-1,4,5,6,7,8-hexahydroquinoline-3-carboxylate (II) Crystal data

**Table:**

C_23_H_27_F_2_NO_4_	*D*_x_ = 1.323 Mg m^−^^3^
*M**_r_* = 419.45	Mo *K*α radiation, λ = 0.71073 Å
Orthorhombic, *P**b**c**a*	Cell parameters from 9744 reflections
*a* = 12.255 (3) Å	θ = 2.3–34.0°
*b* = 15.694 (3) Å	µ = 0.10 mm^−^^1^
*c* = 21.903 (4) Å	*T* = 100 K
*V* = 4212.3 (14) Å^3^	Plate, colorless
*Z* = 8	0.31 × 0.23 × 0.08 mm
*F*(000) = 1776	

### Isopropyl 4-[4-(difluoromethoxy)phenyl]-2,6,6-trimethyl-5-oxo-1,4,5,6,7,8-hexahydroquinoline-3-carboxylate (II) Data collection

**Table:**

Bruker D8 Quest with Photon 2 detector diffractometer	6743 reflections with *I* > 2σ(*I*)
φ and ω scans	*R*_int_ = 0.073
Absorption correction: multi-scan (SADABS; Krause *et al.*, 2015)	θ_max_ = 34.1°, θ_min_ = 2.3°
*T*_min_ = 0.684, *T*_max_ = 0.747	*h* = −15→19
102650 measured reflections	*k* = −24→24
8537 independent reflections	*l* = −32→34

### Isopropyl 4-[4-(difluoromethoxy)phenyl]-2,6,6-trimethyl-5-oxo-1,4,5,6,7,8-hexahydroquinoline-3-carboxylate (II) Refinement

**Table:**

Refinement on *F*^2^	Primary atom site location: dual
Least-squares matrix: full	Secondary atom site location: difference Fourier map
*R*[*F*^2^ > 2σ(*F*^2^)] = 0.050	Hydrogen site location: mixed
*wR*(*F*^2^) = 0.128	H atoms treated by a mixture of independent and constrained refinement
*S* = 1.02	*w* = 1/[σ^2^(*F*_o_^2^) + (0.0602*P*)^2^ + 1.7687*P*] where *P* = (*F*_o_^2^ + 2*F*_c_^2^)/3
8537 reflections	(Δ/σ)_max_ < 0.001
280 parameters	Δρ_max_ = 0.58 e Å^−^^3^
0 restraints	Δρ_min_ = −0.42 e Å^−^^3^

### Isopropyl 4-[4-(difluoromethoxy)phenyl]-2,6,6-trimethyl-5-oxo-1,4,5,6,7,8-hexahydroquinoline-3-carboxylate (II) Special details

**Table:**

Geometry. All esds (except the esd in the dihedral angle between two l.s. planes) are estimated using the full covariance matrix. The cell esds are taken into account individually in the estimation of esds in distances, angles and torsion angles; correlations between esds in cell parameters are only used when they are defined by crystal symmetry. An approximate (isotropic) treatment of cell esds is used for estimating esds involving l.s. planes.

### Isopropyl 4-[4-(difluoromethoxy)phenyl]-2,6,6-trimethyl-5-oxo-1,4,5,6,7,8-hexahydroquinoline-3-carboxylate (II) Fractional atomic coordinates and isotropic or equivalent isotropic displacement parameters (Å^2^)

**Table:**

	*x*	*y*	*z*	*U*_iso_*/*U*_eq_	
F1	0.58006 (8)	0.07640 (5)	0.03801 (3)	0.0364 (2)	
F2	0.64472 (7)	0.00608 (5)	0.11460 (4)	0.0349 (2)	
O1	0.34068 (6)	0.45116 (5)	0.16296 (3)	0.01817 (15)	
O2	0.29601 (6)	0.27319 (5)	0.34751 (4)	0.02216 (16)	
O3	0.41830 (6)	0.29307 (5)	0.42284 (3)	0.01679 (14)	
O4	0.46707 (7)	0.03988 (5)	0.11352 (4)	0.02388 (17)	
N1	0.63612 (7)	0.39398 (5)	0.29383 (4)	0.01310 (14)	
C1	0.59145 (7)	0.43524 (5)	0.24486 (4)	0.01197 (15)	
C2	0.66452 (8)	0.49617 (6)	0.21159 (5)	0.01519 (17)	
H2A	0.708624	0.464675	0.181174	0.018*	
H2B	0.715096	0.523476	0.240904	0.018*	
C3	0.59723 (8)	0.56435 (6)	0.17941 (5)	0.01630 (17)	
H3A	0.645890	0.598521	0.152912	0.020*	
H3B	0.565594	0.603050	0.210406	0.020*	
C4	0.50494 (8)	0.52715 (6)	0.14056 (4)	0.01366 (16)	
C5	0.43666 (8)	0.46417 (6)	0.17775 (4)	0.01252 (16)	
C6	0.48713 (7)	0.41800 (5)	0.22754 (4)	0.01160 (15)	
C7	0.42689 (7)	0.34420 (5)	0.25648 (4)	0.01133 (15)	
H7A	0.347627	0.359096	0.258478	0.014*	
C8	0.46808 (7)	0.33050 (6)	0.32152 (4)	0.01173 (15)	
C9	0.57283 (7)	0.35044 (6)	0.33615 (4)	0.01219 (15)	
C10	0.43280 (9)	0.59982 (7)	0.11768 (6)	0.0233 (2)	
H10A	0.402326	0.630702	0.152635	0.035*	
H10B	0.373259	0.576488	0.092926	0.035*	
H10C	0.476524	0.638908	0.092797	0.035*	
C11	0.55042 (10)	0.47676 (8)	0.08592 (5)	0.0232 (2)	
H11A	0.599642	0.513276	0.062280	0.035*	
H11B	0.489998	0.457985	0.059883	0.035*	
H11C	0.590601	0.426920	0.100808	0.035*	
C12	0.63489 (8)	0.33279 (7)	0.39391 (4)	0.01612 (17)	
H12A	0.595564	0.290247	0.418173	0.024*	
H12B	0.641987	0.385556	0.417509	0.024*	
H12C	0.707624	0.311051	0.383743	0.024*	
C13	0.38614 (8)	0.29573 (6)	0.36382 (4)	0.01362 (16)	
C14	0.34202 (9)	0.25787 (7)	0.46746 (5)	0.0208 (2)	
H14A	0.299229	0.210294	0.448771	0.025*	
C15	0.26612 (14)	0.32793 (11)	0.48827 (7)	0.0461 (4)	
H15A	0.226327	0.350637	0.453038	0.069*	
H15B	0.308701	0.373654	0.507282	0.069*	
H15C	0.214105	0.304965	0.518006	0.069*	
C16	0.41149 (11)	0.22458 (8)	0.51914 (5)	0.0268 (2)	
H16A	0.462584	0.181853	0.503408	0.040*	
H16B	0.364532	0.198499	0.550169	0.040*	
H16C	0.452451	0.271766	0.537407	0.040*	
C17	0.43931 (7)	0.26381 (6)	0.21739 (4)	0.01218 (15)	
C18	0.54172 (8)	0.23882 (6)	0.19612 (5)	0.01650 (17)	
H18A	0.603453	0.272969	0.205585	0.020*	
C19	0.55622 (8)	0.16531 (7)	0.16141 (5)	0.01900 (19)	
H19A	0.626783	0.148978	0.147783	0.023*	
C20	0.46524 (8)	0.11639 (6)	0.14715 (4)	0.01634 (17)	
C21	0.36233 (8)	0.14000 (6)	0.16660 (4)	0.01612 (17)	
H21A	0.300604	0.106507	0.155987	0.019*	
C22	0.34957 (8)	0.21340 (6)	0.20194 (4)	0.01432 (16)	
H22A	0.278885	0.229241	0.215665	0.017*	
C23	0.55424 (9)	0.01649 (7)	0.08023 (5)	0.01990 (19)	
H23A	0.537493	−0.038295	0.058878	0.024*	
H1N	0.7011 (13)	0.4077 (9)	0.3055 (7)	0.020 (3)*	

### Isopropyl 4-[4-(difluoromethoxy)phenyl]-2,6,6-trimethyl-5-oxo-1,4,5,6,7,8-hexahydroquinoline-3-carboxylate (II) Atomic displacement parameters (Å^2^)

**Table:**

	*U*^11^	*U*^22^	*U*^33^	*U*^12^	*U*^13^	*U*^23^
F1	0.0592 (6)	0.0287 (4)	0.0212 (3)	−0.0035 (4)	0.0095 (3)	0.0009 (3)
F2	0.0279 (4)	0.0283 (4)	0.0485 (5)	0.0093 (3)	−0.0177 (3)	−0.0111 (3)
O1	0.0114 (3)	0.0238 (3)	0.0193 (3)	−0.0028 (3)	−0.0020 (3)	0.0071 (3)
O2	0.0142 (3)	0.0315 (4)	0.0207 (3)	−0.0073 (3)	−0.0003 (3)	0.0069 (3)
O3	0.0158 (3)	0.0214 (3)	0.0132 (3)	−0.0012 (3)	0.0028 (2)	0.0032 (2)
O4	0.0220 (4)	0.0201 (3)	0.0295 (4)	−0.0038 (3)	0.0025 (3)	−0.0103 (3)
N1	0.0088 (3)	0.0158 (3)	0.0146 (3)	−0.0017 (3)	−0.0007 (3)	0.0018 (3)
C1	0.0103 (3)	0.0120 (3)	0.0136 (4)	−0.0007 (3)	0.0009 (3)	0.0002 (3)
C2	0.0106 (4)	0.0165 (4)	0.0185 (4)	−0.0031 (3)	0.0004 (3)	0.0036 (3)
C3	0.0162 (4)	0.0141 (4)	0.0187 (4)	−0.0040 (3)	−0.0003 (3)	0.0028 (3)
C4	0.0126 (4)	0.0143 (4)	0.0140 (4)	−0.0014 (3)	0.0010 (3)	0.0032 (3)
C5	0.0116 (4)	0.0129 (3)	0.0130 (4)	−0.0002 (3)	0.0013 (3)	0.0009 (3)
C6	0.0098 (3)	0.0118 (3)	0.0132 (4)	−0.0011 (3)	0.0008 (3)	0.0015 (3)
C7	0.0089 (3)	0.0122 (3)	0.0129 (4)	−0.0013 (3)	0.0004 (3)	0.0014 (3)
C8	0.0106 (3)	0.0124 (3)	0.0122 (4)	−0.0004 (3)	0.0008 (3)	0.0016 (3)
C9	0.0116 (4)	0.0122 (3)	0.0128 (4)	0.0005 (3)	0.0003 (3)	0.0006 (3)
C10	0.0192 (5)	0.0204 (4)	0.0303 (5)	−0.0010 (4)	−0.0028 (4)	0.0115 (4)
C11	0.0246 (5)	0.0276 (5)	0.0173 (4)	−0.0048 (4)	0.0053 (4)	−0.0025 (4)
C12	0.0138 (4)	0.0200 (4)	0.0146 (4)	0.0000 (3)	−0.0025 (3)	0.0018 (3)
C13	0.0130 (4)	0.0132 (3)	0.0146 (4)	0.0004 (3)	0.0017 (3)	0.0026 (3)
C14	0.0191 (5)	0.0250 (5)	0.0182 (4)	0.0016 (4)	0.0068 (4)	0.0083 (4)
C15	0.0432 (8)	0.0545 (9)	0.0405 (7)	0.0299 (7)	0.0263 (7)	0.0257 (7)
C16	0.0316 (6)	0.0323 (6)	0.0167 (4)	0.0083 (5)	0.0048 (4)	0.0073 (4)
C17	0.0115 (4)	0.0127 (3)	0.0123 (4)	−0.0015 (3)	−0.0006 (3)	0.0018 (3)
C18	0.0117 (4)	0.0167 (4)	0.0211 (4)	−0.0031 (3)	0.0006 (3)	−0.0029 (3)
C19	0.0144 (4)	0.0187 (4)	0.0239 (5)	−0.0022 (3)	0.0024 (4)	−0.0052 (4)
C20	0.0176 (4)	0.0152 (4)	0.0162 (4)	−0.0016 (3)	−0.0004 (3)	−0.0025 (3)
C21	0.0142 (4)	0.0173 (4)	0.0169 (4)	−0.0038 (3)	−0.0020 (3)	−0.0009 (3)
C22	0.0113 (4)	0.0159 (4)	0.0158 (4)	−0.0017 (3)	−0.0013 (3)	0.0008 (3)
C23	0.0222 (5)	0.0189 (4)	0.0186 (4)	0.0022 (4)	−0.0028 (4)	−0.0025 (3)

### Isopropyl 4-[4-(difluoromethoxy)phenyl]-2,6,6-trimethyl-5-oxo-1,4,5,6,7,8-hexahydroquinoline-3-carboxylate (II) Geometric parameters (Å, º)

**Table:**

F1—C23	1.3561 (13)	C10—H10A	0.9800
F2—C23	1.3501 (13)	C10—H10B	0.9800
O1—C5	1.2369 (12)	C10—H10C	0.9800
O2—C13	1.2136 (12)	C11—H11A	0.9800
O3—C13	1.3521 (12)	C11—H11B	0.9800
O3—C14	1.4608 (12)	C11—H11C	0.9800
O4—C23	1.3445 (14)	C12—H12A	0.9800
O4—C20	1.4089 (12)	C12—H12B	0.9800
N1—C1	1.3673 (12)	C12—H12C	0.9800
N1—C9	1.3884 (12)	C14—C16	1.5098 (16)
N1—H1N	0.863 (16)	C14—C15	1.5106 (18)
C1—C6	1.3606 (13)	C14—H14A	1.0000
C1—C2	1.4991 (13)	C15—H15A	0.9800
C2—C3	1.5236 (14)	C15—H15B	0.9800
C2—H2A	0.9900	C15—H15C	0.9800
C2—H2B	0.9900	C16—H16A	0.9800
C3—C4	1.5311 (14)	C16—H16B	0.9800
C3—H3A	0.9900	C16—H16C	0.9800
C3—H3B	0.9900	C17—C18	1.3950 (13)
C4—C10	1.5276 (14)	C17—C22	1.3963 (13)
C4—C5	1.5297 (13)	C18—C19	1.3930 (14)
C4—C11	1.5390 (15)	C18—H18A	0.9500
C5—C6	1.4481 (13)	C19—C20	1.3892 (14)
C6—C7	1.5127 (12)	C19—H19A	0.9500
C7—C8	1.5265 (13)	C20—C21	1.3817 (14)
C7—C17	1.5323 (13)	C21—C22	1.3966 (14)
C7—H7A	1.0000	C21—H21A	0.9500
C8—C9	1.3595 (13)	C22—H22A	0.9500
C8—C13	1.4712 (13)	C23—H23A	1.0000
C9—C12	1.5019 (13)		
			
C13—O3—C14	117.69 (8)	H11A—C11—H11C	109.5
C23—O4—C20	121.93 (9)	H11B—C11—H11C	109.5
C1—N1—C9	122.21 (8)	C9—C12—H12A	109.5
C1—N1—H1N	118.8 (10)	C9—C12—H12B	109.5
C9—N1—H1N	116.2 (10)	H12A—C12—H12B	109.5
C6—C1—N1	120.05 (8)	C9—C12—H12C	109.5
C6—C1—C2	123.54 (8)	H12A—C12—H12C	109.5
N1—C1—C2	116.37 (8)	H12B—C12—H12C	109.5
C1—C2—C3	110.46 (8)	O2—C13—O3	122.53 (9)
C1—C2—H2A	109.6	O2—C13—C8	122.95 (9)
C3—C2—H2A	109.6	O3—C13—C8	114.49 (8)
C1—C2—H2B	109.6	O3—C14—C16	105.76 (9)
C3—C2—H2B	109.6	O3—C14—C15	108.70 (9)
H2A—C2—H2B	108.1	C16—C14—C15	111.89 (11)
C2—C3—C4	112.90 (8)	O3—C14—H14A	110.1
C2—C3—H3A	109.0	C16—C14—H14A	110.1
C4—C3—H3A	109.0	C15—C14—H14A	110.1
C2—C3—H3B	109.0	C14—C15—H15A	109.5
C4—C3—H3B	109.0	C14—C15—H15B	109.5
H3A—C3—H3B	107.8	H15A—C15—H15B	109.5
C10—C4—C5	109.91 (8)	C14—C15—H15C	109.5
C10—C4—C3	108.98 (8)	H15A—C15—H15C	109.5
C5—C4—C3	110.77 (8)	H15B—C15—H15C	109.5
C10—C4—C11	109.76 (9)	C14—C16—H16A	109.5
C5—C4—C11	106.27 (8)	C14—C16—H16B	109.5
C3—C4—C11	111.13 (8)	H16A—C16—H16B	109.5
O1—C5—C6	121.38 (8)	C14—C16—H16C	109.5
O1—C5—C4	119.16 (8)	H16A—C16—H16C	109.5
C6—C5—C4	119.38 (8)	H16B—C16—H16C	109.5
C1—C6—C5	120.78 (8)	C18—C17—C22	117.93 (9)
C1—C6—C7	119.60 (8)	C18—C17—C7	120.50 (8)
C5—C6—C7	119.36 (8)	C22—C17—C7	121.58 (8)
C6—C7—C8	109.72 (7)	C19—C18—C17	121.99 (9)
C6—C7—C17	110.35 (7)	C19—C18—H18A	119.0
C8—C7—C17	111.88 (7)	C17—C18—H18A	119.0
C6—C7—H7A	108.3	C20—C19—C18	118.55 (9)
C8—C7—H7A	108.3	C20—C19—H19A	120.7
C17—C7—H7A	108.3	C18—C19—H19A	120.7
C9—C8—C13	125.54 (8)	C21—C20—C19	121.00 (9)
C9—C8—C7	119.98 (8)	C21—C20—O4	113.85 (9)
C13—C8—C7	114.47 (8)	C19—C20—O4	125.15 (9)
C8—C9—N1	118.91 (8)	C20—C21—C22	119.62 (9)
C8—C9—C12	129.36 (8)	C20—C21—H21A	120.2
N1—C9—C12	111.73 (8)	C22—C21—H21A	120.2
C4—C10—H10A	109.5	C17—C22—C21	120.89 (9)
C4—C10—H10B	109.5	C17—C22—H22A	119.6
H10A—C10—H10B	109.5	C21—C22—H22A	119.6
C4—C10—H10C	109.5	O4—C23—F2	112.53 (9)
H10A—C10—H10C	109.5	O4—C23—F1	111.46 (9)
H10B—C10—H10C	109.5	F2—C23—F1	105.81 (10)
C4—C11—H11A	109.5	O4—C23—H23A	109.0
C4—C11—H11B	109.5	F2—C23—H23A	109.0
H11A—C11—H11B	109.5	F1—C23—H23A	109.0
C4—C11—H11C	109.5		
			
C9—N1—C1—C6	−16.13 (13)	C7—C8—C9—N1	8.88 (13)
C9—N1—C1—C2	165.96 (8)	C13—C8—C9—C12	8.63 (16)
C6—C1—C2—C3	27.78 (13)	C7—C8—C9—C12	−171.62 (9)
N1—C1—C2—C3	−154.38 (8)	C1—N1—C9—C8	16.09 (13)
C1—C2—C3—C4	−50.39 (11)	C1—N1—C9—C12	−163.49 (8)
C2—C3—C4—C10	172.05 (8)	C14—O3—C13—O2	2.82 (14)
C2—C3—C4—C5	51.01 (11)	C14—O3—C13—C8	−178.91 (8)
C2—C3—C4—C11	−66.87 (11)	C9—C8—C13—O2	−174.48 (10)
C10—C4—C5—O1	34.21 (12)	C7—C8—C13—O2	5.75 (13)
C3—C4—C5—O1	154.69 (9)	C9—C8—C13—O3	7.26 (13)
C11—C4—C5—O1	−84.49 (11)	C7—C8—C13—O3	−172.51 (8)
C10—C4—C5—C6	−148.80 (9)	C13—O3—C14—C16	153.97 (9)
C3—C4—C5—C6	−28.32 (12)	C13—O3—C14—C15	−85.73 (13)
C11—C4—C5—C6	92.50 (10)	C6—C7—C17—C18	48.03 (11)
N1—C1—C6—C5	176.85 (8)	C8—C7—C17—C18	−74.44 (11)
C2—C1—C6—C5	−5.39 (14)	C6—C7—C17—C22	−132.01 (9)
N1—C1—C6—C7	−9.05 (13)	C8—C7—C17—C22	105.53 (10)
C2—C1—C6—C7	168.71 (8)	C22—C17—C18—C19	−1.13 (15)
O1—C5—C6—C1	−177.45 (9)	C7—C17—C18—C19	178.83 (9)
C4—C5—C6—C1	5.63 (13)	C17—C18—C19—C20	0.81 (16)
O1—C5—C6—C7	8.44 (13)	C18—C19—C20—C21	0.29 (16)
C4—C5—C6—C7	−168.48 (8)	C18—C19—C20—O4	−178.78 (10)
C1—C6—C7—C8	29.90 (11)	C23—O4—C20—C21	165.58 (10)
C5—C6—C7—C8	−155.92 (8)	C23—O4—C20—C19	−15.29 (16)
C1—C6—C7—C17	−93.83 (10)	C19—C20—C21—C22	−1.02 (15)
C5—C6—C7—C17	80.35 (10)	O4—C20—C21—C22	178.15 (9)
C6—C7—C8—C9	−29.89 (11)	C18—C17—C22—C21	0.38 (14)
C17—C7—C8—C9	92.94 (10)	C7—C17—C22—C21	−179.59 (8)
C6—C7—C8—C13	149.89 (8)	C20—C21—C22—C17	0.68 (15)
C17—C7—C8—C13	−87.29 (9)	C20—O4—C23—F2	61.18 (13)
C13—C8—C9—N1	−170.87 (8)	C20—O4—C23—F1	−57.50 (13)

### Isopropyl 4-[4-(difluoromethoxy)phenyl]-2,6,6-trimethyl-5-oxo-1,4,5,6,7,8-hexahydroquinoline-3-carboxylate (II) Hydrogen-bond geometry (Å, º)

**Table:**

*D*—H···*A*	*D*—H	H···*A*	*D*···*A*	*D*—H···*A*
N1—H1*N*···O1^i^	0.863 (16)	1.967 (16)	2.8258 (12)	173.0 (14)
C2—H2*A*···F2^ii^	0.99	2.40	3.1626 (13)	133
C12—H12*A*···O3	0.98	2.18	2.7991 (14)	120
C19—H19*A*···F2	0.95	2.37	2.9106 (14)	116
C23—H23*A*···F1^iii^	1.00	2.63	3.3972 (14)	133

Symmetry codes: (i) *x*+1/2, *y*, −*z*+1/2; (ii) −*x*+3/2, *y*+1/2, *z*; (iii) −*x*+1, −*y*, −*z*.

### tert-Butyl 4-[4-(difluoromethoxy)phenyl]-2,6,6-trimethyl-5-oxo-1,4,5,6,7,8-hexahydroquinoline-3-carboxylate (III) Crystal data

**Table:**

C_24_H_29_F_2_NO_4_	*D*_x_ = 1.322 Mg m^−^^3^
*M**_r_* = 433.48	Mo *K*α radiation, λ = 0.71073 Å
Orthorhombic, *P**b**c**a*	Cell parameters from 8996 reflections
*a* = 12.4094 (8) Å	θ = 2.3–30.3°
*b* = 15.9871 (12) Å	µ = 0.10 mm^−^^1^
*c* = 21.9629 (15) Å	*T* = 100 K
*V* = 4357.2 (5) Å^3^	Plate, colorless
*Z* = 8	0.31 × 0.27 × 0.09 mm
*F*(000) = 1840	

### tert-Butyl 4-[4-(difluoromethoxy)phenyl]-2,6,6-trimethyl-5-oxo-1,4,5,6,7,8-hexahydroquinoline-3-carboxylate (III) Data collection

**Table:**

Bruker APEXII CCD diffractometer	4732 reflections with *I* > 2σ(*I*)
φ and ω scans	*R*_int_ = 0.142
Absorption correction: multi-scan (SADABS; Krause *et al.*, 2015)	θ_max_ = 30.6°, θ_min_ = 1.9°
*T*_min_ = 0.374, *T*_max_ = 0.746	*h* = −17→17
56620 measured reflections	*k* = −22→22
6654 independent reflections	*l* = −31→31

### tert-Butyl 4-[4-(difluoromethoxy)phenyl]-2,6,6-trimethyl-5-oxo-1,4,5,6,7,8-hexahydroquinoline-3-carboxylate (III) Refinement

**Table:**

Refinement on *F*^2^	Primary atom site location: dual
Least-squares matrix: full	Secondary atom site location: difference Fourier map
*R*[*F*^2^ > 2σ(*F*^2^)] = 0.059	Hydrogen site location: mixed
*wR*(*F*^2^) = 0.163	H atoms treated by a mixture of independent and constrained refinement
*S* = 1.05	*w* = 1/[σ^2^(*F*_o_^2^) + (0.0685*P*)^2^ + 0.6147*P*] where *P* = (*F*_o_^2^ + 2*F*_c_^2^)/3
6654 reflections	(Δ/σ)_max_ = 0.001
329 parameters	Δρ_max_ = 0.37 e Å^−^^3^
68 restraints	Δρ_min_ = −0.31 e Å^−^^3^

### tert-Butyl 4-[4-(difluoromethoxy)phenyl]-2,6,6-trimethyl-5-oxo-1,4,5,6,7,8-hexahydroquinoline-3-carboxylate (III) Special details

**Table:**

Geometry. All esds (except the esd in the dihedral angle between two l.s. planes) are estimated using the full covariance matrix. The cell esds are taken into account individually in the estimation of esds in distances, angles and torsion angles; correlations between esds in cell parameters are only used when they are defined by crystal symmetry. An approximate (isotropic) treatment of cell esds is used for estimating esds involving l.s. planes.

### tert-Butyl 4-[4-(difluoromethoxy)phenyl]-2,6,6-trimethyl-5-oxo-1,4,5,6,7,8-hexahydroquinoline-3-carboxylate (III) Fractional atomic coordinates and isotropic or equivalent isotropic displacement parameters (Å^2^)

**Table:**

	*x*	*y*	*z*	*U*_iso_*/*U*_eq_	Occ. (<1)
F1	0.57517 (9)	0.94793 (6)	0.95792 (5)	0.0321 (2)	
F2	0.64034 (9)	1.01340 (7)	0.88027 (6)	0.0401 (3)	
O1	0.32590 (9)	0.56945 (7)	0.83355 (5)	0.0243 (2)	
O2	0.46372 (9)	0.98316 (7)	0.88197 (5)	0.0257 (3)	
O3	0.28837 (10)	0.73942 (9)	0.65077 (6)	0.0347 (3)	
O4	0.41461 (9)	0.72089 (6)	0.57815 (5)	0.0202 (2)	
N1	0.62614 (10)	0.63107 (7)	0.71106 (6)	0.0164 (2)	
C1	0.56462 (11)	0.67209 (8)	0.66740 (6)	0.0153 (3)	
C2	0.46038 (11)	0.69097 (8)	0.68021 (6)	0.0161 (3)	
C3	0.41866 (11)	0.67977 (8)	0.74513 (6)	0.0161 (3)	
H3A	0.340091	0.666165	0.743048	0.019*	
C4	0.47622 (11)	0.60720 (8)	0.77510 (6)	0.0168 (3)	
C5	0.42280 (11)	0.55954 (8)	0.82239 (7)	0.0174 (3)	
C6	0.48853 (12)	0.49691 (8)	0.85970 (7)	0.0185 (3)	
C7	0.58499 (18)	0.46128 (13)	0.82234 (11)	0.0190 (5)	0.646 (3)
H7A	0.632708	0.429090	0.849834	0.023*	0.646 (3)
H7B	0.557191	0.422424	0.790953	0.023*	0.646 (3)
C8	0.6512 (9)	0.5311 (7)	0.7910 (4)	0.0193 (9)	0.646 (3)
H8A	0.694593	0.560970	0.821974	0.023*	0.646 (3)
H8B	0.701396	0.505677	0.761262	0.023*	0.646 (3)
C11	0.5333 (2)	0.54966 (15)	0.91386 (11)	0.0229 (5)	0.646 (3)
H11A	0.571754	0.598568	0.897985	0.034*	0.646 (3)
H11B	0.582927	0.515342	0.937978	0.034*	0.646 (3)
H11C	0.473433	0.568179	0.939625	0.034*	0.646 (3)
C12	0.4219 (2)	0.42518 (15)	0.88388 (12)	0.0263 (6)	0.646 (3)
H12A	0.361872	0.447214	0.907996	0.039*	0.646 (3)
H12B	0.467089	0.389375	0.909586	0.039*	0.646 (3)
H12C	0.393637	0.392387	0.849735	0.039*	0.646 (3)
C7A	0.6013 (3)	0.5198 (3)	0.86174 (19)	0.0187 (8)	0.354 (3)
H7AA	0.641688	0.476110	0.884062	0.022*	0.354 (3)
H7AB	0.609014	0.572916	0.884527	0.022*	0.354 (3)
C8A	0.6504 (16)	0.5304 (12)	0.7986 (7)	0.0181 (13)	0.354 (3)
H8AA	0.654909	0.475285	0.778147	0.022*	0.354 (3)
H8AB	0.724400	0.553046	0.802297	0.022*	0.354 (3)
C11A	0.4353 (4)	0.4888 (3)	0.9231 (2)	0.0263 (10)	0.354 (3)
H11D	0.449832	0.539399	0.946901	0.039*	0.354 (3)
H11E	0.464945	0.440054	0.944246	0.039*	0.354 (3)
H11F	0.357278	0.481932	0.918181	0.039*	0.354 (3)
C12A	0.4654 (4)	0.4132 (3)	0.8248 (2)	0.0243 (9)	0.354 (3)
H12D	0.387682	0.402116	0.824822	0.036*	0.354 (3)
H12E	0.503045	0.367006	0.845007	0.036*	0.354 (3)
H12F	0.490895	0.418052	0.782704	0.036*	0.354 (3)
C9	0.58058 (11)	0.59079 (8)	0.75968 (6)	0.0167 (3)	
C10	0.62756 (12)	0.68867 (9)	0.61031 (7)	0.0207 (3)	
H10A	0.595418	0.735945	0.588470	0.031*	
H10B	0.626017	0.638871	0.584301	0.031*	
H10C	0.702331	0.702011	0.620876	0.031*	
C13	0.37937 (12)	0.72026 (9)	0.63625 (7)	0.0186 (3)	
C14	0.34411 (13)	0.74324 (9)	0.52654 (7)	0.0214 (3)	
C15	0.2548 (2)	0.67935 (14)	0.52089 (12)	0.0622 (8)	
H15A	0.207089	0.683389	0.556308	0.093*	
H15B	0.286095	0.623115	0.519003	0.093*	
H15C	0.213484	0.690132	0.483698	0.093*	
C16	0.4207 (2)	0.73950 (15)	0.47310 (8)	0.0477 (6)	
H16A	0.480326	0.778580	0.479785	0.072*	
H16B	0.382166	0.754898	0.435784	0.072*	
H16C	0.449097	0.682580	0.469071	0.072*	
C17	0.30051 (15)	0.83103 (10)	0.53339 (7)	0.0292 (4)	
H17A	0.360098	0.869678	0.541516	0.044*	
H17B	0.249301	0.832725	0.567338	0.044*	
H17C	0.263853	0.847613	0.495754	0.044*	
C18	0.43219 (11)	0.75974 (9)	0.78268 (6)	0.0168 (3)	
C19	0.34560 (12)	0.81251 (9)	0.79479 (7)	0.0192 (3)	
H19A	0.276094	0.798378	0.779861	0.023*	
C20	0.35903 (12)	0.88595 (9)	0.82852 (7)	0.0213 (3)	
H20A	0.299016	0.920984	0.836923	0.026*	
C21	0.46028 (12)	0.90708 (9)	0.84950 (7)	0.0192 (3)	
C22	0.54853 (13)	0.85619 (10)	0.83835 (7)	0.0247 (3)	
H22A	0.618017	0.871002	0.852956	0.030*	
C23	0.53307 (12)	0.78277 (10)	0.80522 (7)	0.0229 (3)	
H23A	0.593071	0.747316	0.797753	0.028*	
C24	0.55049 (13)	1.00616 (9)	0.91461 (7)	0.0233 (3)	
H24A	0.535474	1.060914	0.934921	0.028*	
H1N	0.6911 (17)	0.6159 (12)	0.6999 (9)	0.030 (5)*	

### tert-Butyl 4-[4-(difluoromethoxy)phenyl]-2,6,6-trimethyl-5-oxo-1,4,5,6,7,8-hexahydroquinoline-3-carboxylate (III) Atomic displacement parameters (Å^2^)

**Table:**

	*U*^11^	*U*^22^	*U*^33^	*U*^12^	*U*^13^	*U*^23^
F1	0.0440 (6)	0.0243 (5)	0.0281 (5)	0.0072 (4)	−0.0078 (5)	−0.0008 (4)
F2	0.0317 (6)	0.0286 (5)	0.0601 (7)	−0.0069 (4)	0.0200 (5)	−0.0048 (5)
O1	0.0156 (5)	0.0303 (6)	0.0270 (6)	0.0012 (4)	0.0020 (5)	0.0096 (4)
O2	0.0261 (6)	0.0196 (5)	0.0313 (6)	0.0068 (4)	−0.0035 (5)	−0.0056 (4)
O3	0.0185 (6)	0.0584 (8)	0.0272 (6)	0.0125 (5)	0.0026 (5)	0.0157 (6)
O4	0.0201 (5)	0.0228 (5)	0.0176 (5)	0.0039 (4)	−0.0023 (4)	0.0010 (4)
N1	0.0112 (5)	0.0181 (5)	0.0199 (6)	0.0011 (4)	0.0002 (5)	0.0010 (4)
C1	0.0147 (6)	0.0137 (6)	0.0175 (6)	−0.0006 (5)	−0.0017 (5)	−0.0001 (5)
C2	0.0155 (6)	0.0143 (6)	0.0186 (6)	−0.0001 (5)	−0.0007 (5)	0.0019 (5)
C3	0.0121 (6)	0.0179 (6)	0.0184 (6)	0.0020 (5)	−0.0003 (5)	0.0022 (5)
C4	0.0136 (6)	0.0175 (6)	0.0194 (6)	0.0003 (5)	−0.0028 (6)	0.0035 (5)
C5	0.0154 (6)	0.0176 (6)	0.0193 (6)	−0.0006 (5)	−0.0023 (6)	0.0019 (5)
C6	0.0193 (7)	0.0162 (6)	0.0201 (7)	0.0014 (5)	−0.0006 (6)	0.0034 (5)
C7	0.0182 (10)	0.0158 (9)	0.0231 (10)	0.0033 (7)	−0.0011 (9)	0.0010 (7)
C8	0.0145 (14)	0.0217 (14)	0.022 (2)	−0.0021 (12)	−0.0006 (15)	0.0053 (15)
C11	0.0241 (12)	0.0249 (11)	0.0195 (10)	0.0039 (9)	−0.0038 (10)	0.0001 (8)
C12	0.0198 (11)	0.0246 (11)	0.0345 (13)	−0.0014 (9)	0.0000 (10)	0.0132 (10)
C7A	0.0147 (15)	0.0200 (15)	0.0213 (16)	0.0008 (13)	−0.0055 (14)	0.0033 (13)
C8A	0.013 (2)	0.018 (2)	0.023 (3)	0.007 (2)	−0.003 (2)	0.008 (2)
C11A	0.024 (2)	0.032 (2)	0.023 (2)	0.0093 (17)	0.0002 (18)	0.0048 (17)
C12A	0.024 (2)	0.0181 (18)	0.031 (2)	−0.0044 (15)	0.0053 (18)	−0.0015 (16)
C9	0.0146 (6)	0.0153 (6)	0.0203 (7)	−0.0006 (5)	−0.0022 (6)	0.0015 (5)
C10	0.0172 (7)	0.0236 (7)	0.0212 (7)	−0.0008 (5)	0.0019 (6)	0.0012 (5)
C13	0.0162 (6)	0.0203 (6)	0.0192 (7)	−0.0002 (5)	−0.0008 (6)	0.0039 (5)
C14	0.0269 (8)	0.0177 (6)	0.0196 (7)	0.0011 (6)	−0.0087 (6)	0.0010 (5)
C15	0.0774 (17)	0.0428 (11)	0.0665 (15)	−0.0359 (12)	−0.0534 (14)	0.0279 (11)
C16	0.0590 (14)	0.0649 (13)	0.0190 (8)	0.0378 (11)	−0.0010 (9)	−0.0003 (8)
C17	0.0353 (9)	0.0287 (8)	0.0236 (7)	0.0132 (7)	−0.0059 (7)	−0.0002 (6)
C18	0.0151 (7)	0.0198 (6)	0.0156 (6)	0.0018 (5)	0.0014 (5)	0.0022 (5)
C19	0.0141 (6)	0.0197 (6)	0.0239 (7)	0.0018 (5)	0.0007 (6)	0.0038 (5)
C20	0.0183 (7)	0.0193 (6)	0.0262 (7)	0.0055 (5)	0.0034 (6)	0.0026 (5)
C21	0.0218 (7)	0.0177 (6)	0.0181 (6)	0.0029 (5)	0.0011 (6)	0.0005 (5)
C22	0.0172 (7)	0.0293 (8)	0.0274 (8)	0.0046 (6)	−0.0044 (6)	−0.0068 (6)
C23	0.0164 (7)	0.0269 (7)	0.0255 (7)	0.0063 (6)	−0.0009 (6)	−0.0073 (6)
C24	0.0223 (7)	0.0178 (6)	0.0299 (8)	0.0001 (5)	0.0027 (7)	−0.0004 (6)

### tert-Butyl 4-[4-(difluoromethoxy)phenyl]-2,6,6-trimethyl-5-oxo-1,4,5,6,7,8-hexahydroquinoline-3-carboxylate (III) Geometric parameters (Å, º)

**Table:**

F1—C24	1.3658 (18)	C7A—C8A	1.525 (16)
F2—C24	1.3510 (19)	C7A—H7AA	0.9900
O1—C5	1.2374 (18)	C7A—H7AB	0.9900
O2—C24	1.3449 (19)	C8A—C9	1.553 (18)
O2—C21	1.4107 (17)	C8A—H8AA	0.9900
O3—C13	1.2127 (19)	C8A—H8AB	0.9900
O4—C13	1.3490 (18)	C11A—H11D	0.9800
O4—C14	1.4757 (17)	C11A—H11E	0.9800
N1—C9	1.3692 (18)	C11A—H11F	0.9800
N1—C1	1.3902 (18)	C12A—H12D	0.9800
N1—H1N	0.88 (2)	C12A—H12E	0.9800
C1—C2	1.3578 (19)	C12A—H12F	0.9800
C1—C10	1.501 (2)	C10—H10A	0.9800
C2—C13	1.470 (2)	C10—H10B	0.9800
C2—C3	1.5273 (19)	C10—H10C	0.9800
C3—C4	1.5132 (18)	C14—C16	1.511 (3)
C3—C18	1.531 (2)	C14—C17	1.512 (2)
C3—H3A	1.0000	C14—C15	1.512 (2)
C4—C9	1.364 (2)	C15—H15A	0.9800
C4—C5	1.449 (2)	C15—H15B	0.9800
C5—C6	1.5295 (19)	C15—H15C	0.9800
C6—C7A	1.447 (4)	C16—H16A	0.9800
C6—C12	1.510 (3)	C16—H16B	0.9800
C6—C11A	1.546 (5)	C16—H16C	0.9800
C6—C7	1.559 (3)	C17—H17A	0.9800
C6—C11	1.560 (3)	C17—H17B	0.9800
C6—C12A	1.570 (4)	C17—H17C	0.9800
C7—C8	1.547 (10)	C18—C19	1.3918 (19)
C7—H7A	0.9900	C18—C23	1.396 (2)
C7—H7B	0.9900	C19—C20	1.398 (2)
C8—C9	1.468 (11)	C19—H19A	0.9500
C8—H8A	0.9900	C20—C21	1.380 (2)
C8—H8B	0.9900	C20—H20A	0.9500
C11—H11A	0.9800	C21—C22	1.386 (2)
C11—H11B	0.9800	C22—C23	1.394 (2)
C11—H11C	0.9800	C22—H22A	0.9500
C12—H12A	0.9800	C23—H23A	0.9500
C12—H12B	0.9800	C24—H24A	1.0000
C12—H12C	0.9800		
			
C24—O2—C21	121.96 (12)	H11D—C11A—H11E	109.5
C13—O4—C14	122.42 (12)	C6—C11A—H11F	109.5
C9—N1—C1	122.21 (12)	H11D—C11A—H11F	109.5
C9—N1—H1N	117.9 (13)	H11E—C11A—H11F	109.5
C1—N1—H1N	116.2 (13)	C6—C12A—H12D	109.5
C2—C1—N1	119.02 (13)	C6—C12A—H12E	109.5
C2—C1—C10	129.03 (13)	H12D—C12A—H12E	109.5
N1—C1—C10	111.95 (12)	C6—C12A—H12F	109.5
C1—C2—C13	125.87 (13)	H12D—C12A—H12F	109.5
C1—C2—C3	119.37 (12)	H12E—C12A—H12F	109.5
C13—C2—C3	114.74 (12)	C4—C9—N1	119.68 (13)
C4—C3—C2	109.63 (11)	C4—C9—C8	125.1 (4)
C4—C3—C18	110.75 (11)	N1—C9—C8	115.2 (4)
C2—C3—C18	111.59 (11)	C4—C9—C8A	120.9 (7)
C4—C3—H3A	108.3	N1—C9—C8A	119.4 (7)
C2—C3—H3A	108.3	C1—C10—H10A	109.5
C18—C3—H3A	108.3	C1—C10—H10B	109.5
C9—C4—C5	120.75 (13)	H10A—C10—H10B	109.5
C9—C4—C3	119.19 (12)	C1—C10—H10C	109.5
C5—C4—C3	119.94 (12)	H10A—C10—H10C	109.5
O1—C5—C4	121.30 (13)	H10B—C10—H10C	109.5
O1—C5—C6	119.73 (13)	O3—C13—O4	123.28 (14)
C4—C5—C6	118.97 (12)	O3—C13—C2	122.98 (14)
C7A—C6—C5	111.51 (19)	O4—C13—C2	113.70 (12)
C12—C6—C5	113.18 (14)	O4—C14—C16	102.37 (13)
C7A—C6—C11A	114.0 (3)	O4—C14—C17	111.14 (12)
C5—C6—C11A	107.99 (19)	C16—C14—C17	109.82 (14)
C12—C6—C7	109.13 (15)	O4—C14—C15	109.50 (13)
C5—C6—C7	111.51 (13)	C16—C14—C15	111.71 (19)
C12—C6—C11	109.71 (17)	C17—C14—C15	111.91 (17)
C5—C6—C11	104.16 (13)	C14—C15—H15A	109.5
C7—C6—C11	108.97 (16)	C14—C15—H15B	109.5
C7A—C6—C12A	114.1 (3)	H15A—C15—H15B	109.5
C5—C6—C12A	101.5 (2)	C14—C15—H15C	109.5
C11A—C6—C12A	106.9 (3)	H15A—C15—H15C	109.5
C8—C7—C6	112.2 (4)	H15B—C15—H15C	109.5
C8—C7—H7A	109.2	C14—C16—H16A	109.5
C6—C7—H7A	109.2	C14—C16—H16B	109.5
C8—C7—H7B	109.2	H16A—C16—H16B	109.5
C6—C7—H7B	109.2	C14—C16—H16C	109.5
H7A—C7—H7B	107.9	H16A—C16—H16C	109.5
C9—C8—C7	111.1 (7)	H16B—C16—H16C	109.5
C9—C8—H8A	109.4	C14—C17—H17A	109.5
C7—C8—H8A	109.4	C14—C17—H17B	109.5
C9—C8—H8B	109.4	H17A—C17—H17B	109.5
C7—C8—H8B	109.4	C14—C17—H17C	109.5
H8A—C8—H8B	108.0	H17A—C17—H17C	109.5
C6—C11—H11A	109.5	H17B—C17—H17C	109.5
C6—C11—H11B	109.5	C19—C18—C23	117.70 (13)
H11A—C11—H11B	109.5	C19—C18—C3	121.64 (13)
C6—C11—H11C	109.5	C23—C18—C3	120.65 (12)
H11A—C11—H11C	109.5	C18—C19—C20	121.21 (14)
H11B—C11—H11C	109.5	C18—C19—H19A	119.4
C6—C12—H12A	109.5	C20—C19—H19A	119.4
C6—C12—H12B	109.5	C21—C20—C19	119.40 (13)
H12A—C12—H12B	109.5	C21—C20—H20A	120.3
C6—C12—H12C	109.5	C19—C20—H20A	120.3
H12A—C12—H12C	109.5	C20—C21—C22	121.11 (14)
H12B—C12—H12C	109.5	C20—C21—O2	114.03 (13)
C6—C7A—C8A	112.7 (8)	C22—C21—O2	124.86 (14)
C6—C7A—H7AA	109.0	C21—C22—C23	118.53 (14)
C8A—C7A—H7AA	109.0	C21—C22—H22A	120.7
C6—C7A—H7AB	109.0	C23—C22—H22A	120.7
C8A—C7A—H7AB	109.0	C22—C23—C18	122.05 (14)
H7AA—C7A—H7AB	107.8	C22—C23—H23A	119.0
C7A—C8A—C9	110.2 (11)	C18—C23—H23A	119.0
C7A—C8A—H8AA	109.6	O2—C24—F2	112.74 (14)
C9—C8A—H8AA	109.6	O2—C24—F1	111.38 (12)
C7A—C8A—H8AB	109.6	F2—C24—F1	105.20 (13)
C9—C8A—H8AB	109.6	O2—C24—H24A	109.1
H8AA—C8A—H8AB	108.1	F2—C24—H24A	109.1
C6—C11A—H11D	109.5	F1—C24—H24A	109.1
C6—C11A—H11E	109.5		
			
C9—N1—C1—C2	16.6 (2)	C3—C4—C9—N1	−10.2 (2)
C9—N1—C1—C10	−162.96 (12)	C5—C4—C9—C8	−5.5 (5)
N1—C1—C2—C13	−168.52 (13)	C3—C4—C9—C8	170.5 (5)
C10—C1—C2—C13	10.9 (2)	C5—C4—C9—C8A	−9.4 (8)
N1—C1—C2—C3	9.82 (19)	C3—C4—C9—C8A	166.6 (8)
C10—C1—C2—C3	−170.73 (13)	C1—N1—C9—C4	−16.5 (2)
C1—C2—C3—C4	−32.19 (17)	C1—N1—C9—C8	162.9 (4)
C13—C2—C3—C4	146.33 (12)	C1—N1—C9—C8A	166.7 (8)
C1—C2—C3—C18	90.88 (15)	C7—C8—C9—C4	26.3 (8)
C13—C2—C3—C18	−90.60 (14)	C7—C8—C9—N1	−153.0 (4)
C2—C3—C4—C9	32.34 (18)	C7A—C8A—C9—C4	−19.0 (15)
C18—C3—C4—C9	−91.22 (16)	C7A—C8A—C9—N1	157.8 (7)
C2—C3—C4—C5	−151.65 (13)	C14—O4—C13—O3	−1.5 (2)
C18—C3—C4—C5	84.79 (16)	C14—O4—C13—C2	176.23 (12)
C9—C4—C5—O1	−174.39 (14)	C1—C2—C13—O3	−176.94 (15)
C3—C4—C5—O1	9.7 (2)	C3—C2—C13—O3	4.6 (2)
C9—C4—C5—C6	6.6 (2)	C1—C2—C13—O4	5.3 (2)
C3—C4—C5—C6	−169.33 (12)	C3—C2—C13—O4	−173.09 (12)
O1—C5—C6—C7A	−152.9 (2)	C13—O4—C14—C16	176.95 (15)
C4—C5—C6—C7A	26.1 (2)	C13—O4—C14—C17	59.76 (18)
O1—C5—C6—C12	28.8 (2)	C13—O4—C14—C15	−64.4 (2)
C4—C5—C6—C12	−152.19 (17)	C4—C3—C18—C19	−134.72 (14)
O1—C5—C6—C11A	−26.9 (3)	C2—C3—C18—C19	102.85 (15)
C4—C5—C6—C11A	152.1 (2)	C4—C3—C18—C23	46.27 (18)
O1—C5—C6—C7	152.31 (15)	C2—C3—C18—C23	−76.16 (16)
C4—C5—C6—C7	−28.68 (19)	C23—C18—C19—C20	−0.2 (2)
O1—C5—C6—C11	−90.31 (18)	C3—C18—C19—C20	−179.23 (13)
C4—C5—C6—C11	88.70 (17)	C18—C19—C20—C21	0.9 (2)
O1—C5—C6—C12A	85.2 (2)	C19—C20—C21—C22	−0.8 (2)
C4—C5—C6—C12A	−95.7 (2)	C19—C20—C21—O2	178.96 (13)
C12—C6—C7—C8	174.7 (4)	C24—O2—C21—C20	168.08 (14)
C5—C6—C7—C8	48.9 (4)	C24—O2—C21—C22	−12.1 (2)
C11—C6—C7—C8	−65.5 (4)	C20—C21—C22—C23	0.1 (2)
C6—C7—C8—C9	−47.3 (6)	O2—C21—C22—C23	−179.67 (14)
C5—C6—C7A—C8A	−55.3 (8)	C21—C22—C23—C18	0.6 (2)
C11A—C6—C7A—C8A	−177.9 (8)	C19—C18—C23—C22	−0.6 (2)
C12A—C6—C7A—C8A	58.9 (9)	C3—C18—C23—C22	178.48 (14)
C6—C7A—C8A—C9	52.0 (13)	C21—O2—C24—F2	60.27 (18)
C5—C4—C9—N1	173.81 (13)	C21—O2—C24—F1	−57.73 (18)

### tert-Butyl 4-[4-(difluoromethoxy)phenyl]-2,6,6-trimethyl-5-oxo-1,4,5,6,7,8-hexahydroquinoline-3-carboxylate (III) Hydrogen-bond geometry (Å, º)

**Table:**

*D*—H···*A*	*D*—H	H···*A*	*D*···*A*	*D*—H···*A*
N1—H1*N*···O1^i^	0.88 (2)	1.97 (2)	2.8418 (16)	171.2 (19)
C8*A*—H8*A*···F2^ii^	0.99	2.53	3.168 (19)	130
C8AA—H8*AB*···F2^ii^	0.99	2.48	3.168 (19)	126
C10—H10*A*···O4	0.98	2.27	2.7834 (18)	112
C15—H15*A*···O3	0.98	2.47	3.038 (3)	116
C16—H16*C*···F1^iii^	0.98	2.62	3.573 (2)	164
C17—H17*B*···O3	0.98	2.41	2.969 (2)	116
C22—H22*A*···F2	0.95	2.37	2.9091 (19)	116
C24—H24*A*···O4^iv^	1.00	2.65	3.4638 (18)	139

Symmetry codes: (i) *x*+1/2, *y*, −*z*+3/2; (ii) −*x*+3/2, *y*−1/2, *z*; (iii) *x*, −*y*+3/2, *z*−1/2; (iv) −*x*+1, *y*+1/2, −*z*+3/2.
